# Water and nitrogen application enhance eggplant productivity and nitrogen use efficiency in an arid environment

**DOI:** 10.3389/fpls.2026.1878835

**Published:** 2026-08-04

**Authors:** Chenli Zhou, Hengjia Zhang, Xietian Chen, Fuqiang Li, Yan Zhao

**Affiliations:** 1 College of Agricultural and Biology, Liaocheng University, Liaocheng, China; 2 College of Water Conservancy and Hydropower Engineering, Gansu Agricultural University, Lanzhou, China

**Keywords:** dry matter accumulation, eggplant yield, plant growth, resource use efficiency, sustainable production, water-nitrogen management

## Abstract

Water scarcity and excessive nitrogen fertilizer application are bottlenecks constraining the sustainable development of agriculture in the cold and arid Hexi Corridor region of China. However, the interactive effects of deficit irrigation and nitrogen rate on eggplant physiology, yield, and resource efficiency remain insufficiently quantified for this region, and the optimal water–nitrogen combination for practical application is unclear. To address this gap, a two-year field experiment was conducted with three water deficit levels (W0, full irrigation; W1, mild water deficit; and W2, moderate water deficit) and three nitrogen application rates (N1, low nitrogen application; N2, medium nitrogen application; and N3, high nitrogen application). Full irrigation without nitrogen application served as the control. Plant height, stem diameter, leaf area index, and dry matter accumulation (DMA) increased with increasing nitrogen application rates under the W0 and W1 levels. Appropriate water–nitrogen management enhanced DMA in fruits and improved individual fruit number and weight. The W1N2 treatment (soil moisture content at 60–70% field capacity and nitrogen application rate of 270 kg·ha^-1^) achieved the highest yield, crop water productivity, and irrigation water productivity. Compared with the CK, the W1N2 treatment significantly increased (*P* < 0.05) yield, crop water productivity, and irrigation water productivity by 32.62%, 37.84%, and 38.33% in 2021, and by 35.06%, 42.48%, and 45.53% in 2022, respectively. The N3 treatment significantly reduced nitrogen partial factor productivity. In summary, the combination of mild water deficit and medium nitrogen application represents the optimal water–nitrogen management strategy for eggplant production in the Hexi Corridor. This study offers theoretical and technical support for water and nitrogen conservation in sustainable eggplant production in arid regions.

## 1 Introduction

The sustainable development of agriculture in arid and semi-arid regions is critically constrained by the escalating conflict between limited water resources and the demand for intensified agricultural production (Du et al., 2018; Zhou et al., 2024; Muhirwa et al., 2025). The Hexi Corridor, located at Minle County in Gansu Province of China, epitomizes this challenge. Characterized by an extreme low rainfall, high evaporation rates, and fragile ecosystems, the region’s agriculture relies heavily on irrigation (Zhou et al., 2023). However, intensive agricultural practices, particularly for high-value cash crops, often lead to excessive irrigation and over-application of nitrogen fertilizers. This not only compromises water and nitrogen use efficiencies but also triggers severe environmental issues, including groundwater over-extraction, soil salinization, and nitrate pollution (Azad et al., 2020; Fan et al., 2024; Scherger et al., 2021). Therefore, formulating scientific and reasonable irrigation and fertilization strategies is essential to support the long−term sustainable development of agriculture in this region.

Eggplant (*Solanum melongena L*.) is a widely cultivated vegetable crop of significant economic value (Toppino et al., 2020; Karimi et al., 2021). In the Hexi Corridor region, eggplant is one of the primary income sources for local farmers. Similar to most crops, the growth, photosynthetic properties, yield, and quality of eggplants are highly sensitivity to water and nitrogen supply. Currently, local irrigation and fertilization schedules for eggplant are predominantly experience-based, frequently resulting in excessive “flooding and heavy fertilization” practices. Such empirical management not only wastes scarce water resources but also leads to nitrogen leaching, fruit malformation, and reduced quality, undermining both profitability and environmental sustainability.

Against this backdrop, deficit irrigation has gradually emerged as a promising water-saving irrigation method. Studies on tomato has indicated that moderate deficit irrigation presents a valuable trade-off, in which a potential yield reduction is offset by a significant increase in crop water productivity (*WP_C_*) and improvement in fruit quality, including increases in vitamin C, total soluble solids, soluble sugar content, and organic acids (Li et al., 2021). However, severe deficit irrigation alters the chemical equilibrium in the rhizosphere, inhibiting plant physiological activities and overall growth, thereby affecting fruit yield and quality (Coyago-Cruz et al., 2019; He et al., 2021; Hou et al., 2019). Research has indicated that the optimal irrigation rate is not equivalent to the maximum irrigation volume (dos Santos Silva et al., 2021). Oversupply of irrigation diminishes *WP_C_* and may weaken the reactive oxygen species-scavenging mechanisms of the plant, which is detrimental to fruit yield and quality (Ding et al., 2025; Liu et al., 2023; Wang H et al., 2024; Yang et al., 2017). Therefore, the establishment of a rational irrigation system is vital for achieving a balance among water conservation, increased production, and high quality.

In addition to moisture conditions, nitrogen serves as a critical factor influencing crop growth, yield formation, and quality composition (Zhao et al., 2020). However, the application rate of nitrogen must be precisely controlled; otherwise, it results in low utilization efficiency and imposes environmental burdens (Kunrath et al., 2018; Ye et al., 2022; Zamljen et al., 2022). Inadequate nitrogen supply directly restricts plant growth, plant development, yield accumulation, and nitrogen fertilizer utilization efficiency (Li et al., 2021). Excessive nitrogen application fails to enhance yield targets and reduces crop efficiency in utilizing water and fertilizer resources, thereby adversely affecting yield, quality, and nitrogen use efficiency (Cheng et al., 2021; Li et al., 2024; Xu et al., 2023). Excessive nitrogen also causes nitrate leaching, increasing the potential for groundwater contamination, and it weakens the activity of soil microorganisms, thereby affecting the ecological sustainability of agricultural systems (Chen et al., 2025; Lu et al., 2021; Sun et al., 2013).

There is a close physiological and ecological interaction between irrigation and nitrogen fertilizer application (Hou et al., 2024; Xu et al., 2018). Water status directly affects the nitrogen transformation process in the soil and crop nitrogen absorption efficiency, while nitrogen nutrition modulates stomatal conductance and photosynthetic enzyme activity, thereby influencing crop transpiration and water use efficiency (Ye et al., 2024). Thus, an imbalance in either resource inevitably compromises the efficiency of the other (Hou et al., 2024; Mwinuka et al., 2021). For instance, water shortage can drastically reduce nitrogen availability and uptake (Du et al., 2018), appropriate nitrogen fertilizer supply enhances the drought resistance of plants under drought conditions (Zhou et al., 2025; Zhu et al., 2025), while excessive irrigation coupled with excessive nitrogen inputs significantly increases nitrogen leaching losses (Hu et al., 2021; Lv et al., 2019; Scherger et al., 2021). Understanding this synergy is crucial for optimizing resource inputs. However, the specific thresholds and interactive effects governing this water-nitrogen synergy in eggplant, particularly under the unique climatic and edaphic constraints of the Hexi Corridor, remain largely unexplored.

Despite abundant research findings on water-nitrogen interactions in Solanaceous crops (such as tomatoes, peppers, and potatoes), the findings are not directly transferable to eggplants in the Hexi Corridor for several reasons. First, the main root system of eggplants is mostly concentrated in the top layer of the soil, which makes their process of water and nutrient uptake dynamics more sensitive to soil moisture fluctuations. Second, the extreme aridity, high evapotranspiration demand, and alkaline, low-organic-matter soils of the Hexi Corridor create a vastly different environment from the semi-arid or humid regions where most previous studies were conducted. These unique conditions profoundly affect water infiltration, nitrogen transformation, and the physiological responses of the crop. Consequently, directly applying management strategies developed for other regions or other solanaceous crops is unlikely to achieve optimal outcomes and may even be counterproductive.

Therefore, the scientific rationale of this study is to fill this critical knowledge gap by systematically investigating the response of eggplant to combined water and nitrogen management in the Hexi Corridor. We hypothesized that a mild water deficit combined with moderate nitrogen supply maximizes yield and resource use efficiency by balancing vegetative growth and fruit development. To test this hypothesis, this study was conducted with two primary objectives: (1) to evaluate the effects of different irrigation amounts and nitrogen fertilizer application rates on eggplant growth, yield, *WP_C_*, and nitrogen fertilizer use efficiency; and (2) to establish the optimal combination of water and nitrogen fertilizer application for eggplant production in the Hexi Corridor region of Northwest China. By integrating field experiments with comprehensive evaluation methods, the goal of the present study was to provide scientific evidence for enhancing eggplant yield and resource utilization efficiency in arid regions, thereby supporting the sustainable intensive agricultural development of Northwest China.

## 2 Materials and methods

### 2.1 Study site description

**Figure 1:**
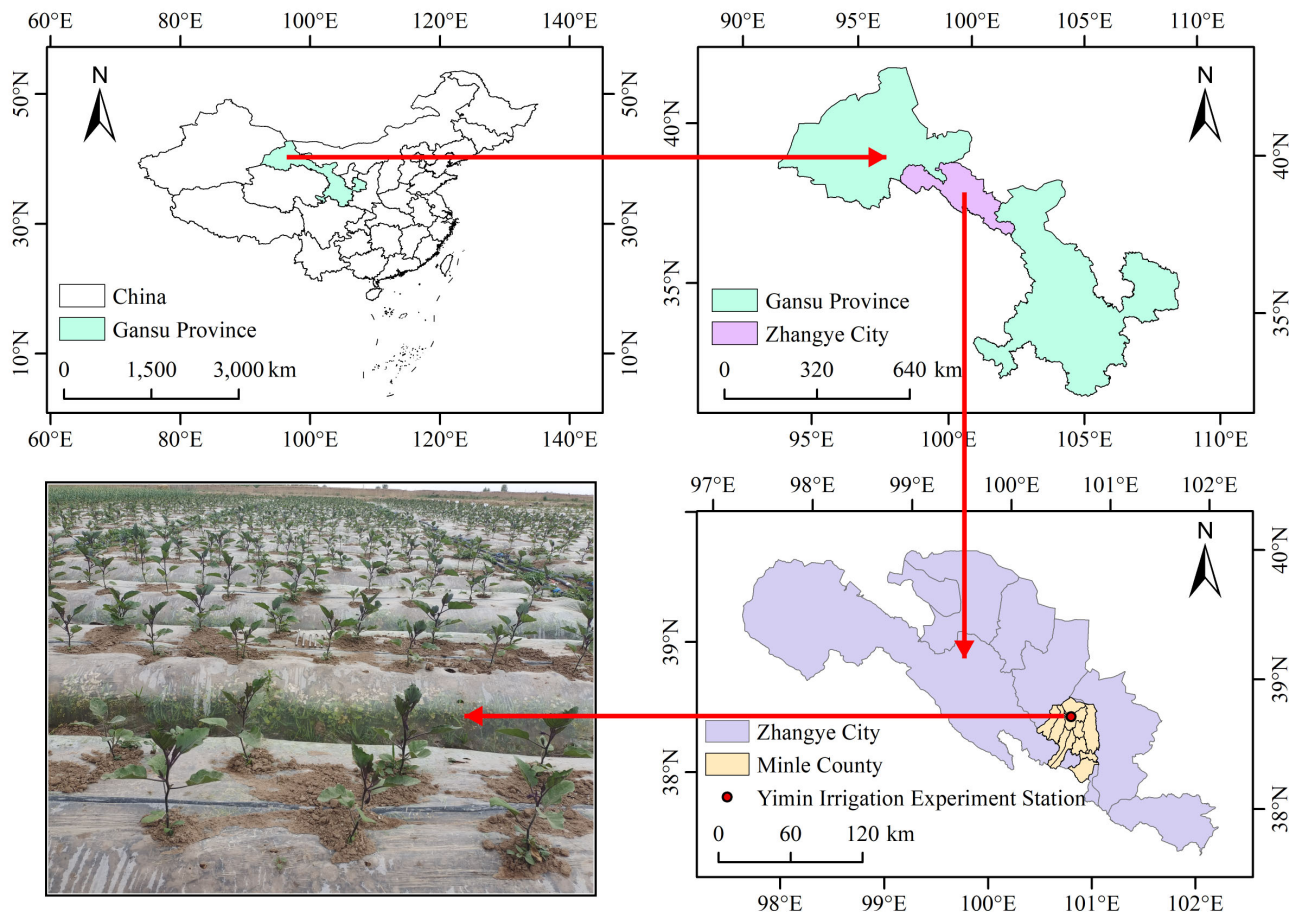
Geographical location map of the experimental area.

Field experiments were performed from May to August in 2021 and 2022 at the Yimin Irrigation Experiment Station (100°43′E and 38°39′N) located in Minle County, Zhangye City, Gansu Province, China (**Figure 1**). The region is characterized by a continental desert steppe climate. Data collected by the Minle County Meteorological Station indicates that over the past 20 years, the multiyear average precipitation and evaporation were approximately 200 mm and 1638 mm, respectively. The annual average temperature, duration of sunshine, aridity index, and frost-free period are 7.6 °C, 2932 hours, 5.85, and 150 days, respectively. The experimental site features light loam soil with moderate fertility. The bulk density of the soil layer within 0–60 cm depth is approximately 1.46 g·cm^-3^, and the field capacity of the 0–100 cm layer is approximately 24% (mass water content). The groundwater table in this area is buried at a depth exceeding 20 meters, and upward recharge is negligible.

### 2.2 Experimental design and field management

The field experiments involved a two-factor experiment combining irrigation and nitrogen application. The irrigation levels were set at the following three gradients: fully irrigated (W0), mild water deficit (W1), and moderate water deficit (W2), with soil moisture contents at 70–80%, 60–70%, and 50–60% of field capacity, respectively. Nitrogen application rates were established across the following three gradients: low nitrogen (N1, nitrogen fertilizer application rate of 215 kg·ha^-1^), medium nitrogen (N2, nitrogen fertilizer application rate of 270 kg·ha^-1^), and high nitrogen (N3, nitrogen fertilizer application rate of 325 kg·ha^-1^). Based on the growth and development of eggplant plants, the entire growing season of eggplant plants is classified into four distinct stages based on their developmental status as follows: seedling period (May 9 to June 3 in 2021; May 11 to June 3 in 2022); flowering and fruit set period (June 4 to July 5 in 2021; June 4 to July 5 in 2022); fruit development period (July 6 to August 2 in 2021; July 6 to August 2 in 2022); and the fruit ripening period (August 3 to August 28 in 2021; August 3 to August 30 in 2022). Only mild and moderate water deficit management was imposed during the flowering and fruit set period, while adequate irrigation was provided during the other growth stages. The control (CK) treatment consisted of a full water supply throughout the entire growth period (W0) and no nitrogen supply (N0). A total of ten treatments were established, including nine treatment involving water–nitrogen regulation and one control treatment. Three replicates were conducted for each treatment, with a total of 30 plots. The specific experimental design is shown in **Table 1**.

**Table 1:** Experimental design.

Treatments	Soil moisture level				Nitrogen application rate (kg·ha^-1^)
	Seedling	Flowering and fruit set	Fruit development	Fruit ripening	
CK	70–80%^a^	70–80%	70–80%	70–80%	0
W_0_ N_1_	70–80%	70–80%	70–80%	70–80%	215
W_0_ N_2_	70–80%	70–80%	70–80%	70–80%	270
W_0_ N_3_	70–80%	70–80%	70–80%	70–80%	325
W_1_ N_1_	70–80%	60–70%	70–80%	70–80%	215
W_1_ N_2_	70–80%	60–70%	70–80%	70–80%	270
W_1_ N_3_	70–80%	60–70%	70–80%	70–80%	325
W_2_ N_1_	70–80%	50–60%	70–80%	70–80%	215
W_2_ N_2_	70–80%	50–60%	70–80%	70–80%	270
W2 N3	70–80%	50–60%	70–80%	70–80%	325

^a^ percentage in field capacity (FC).

**Figure 2:**
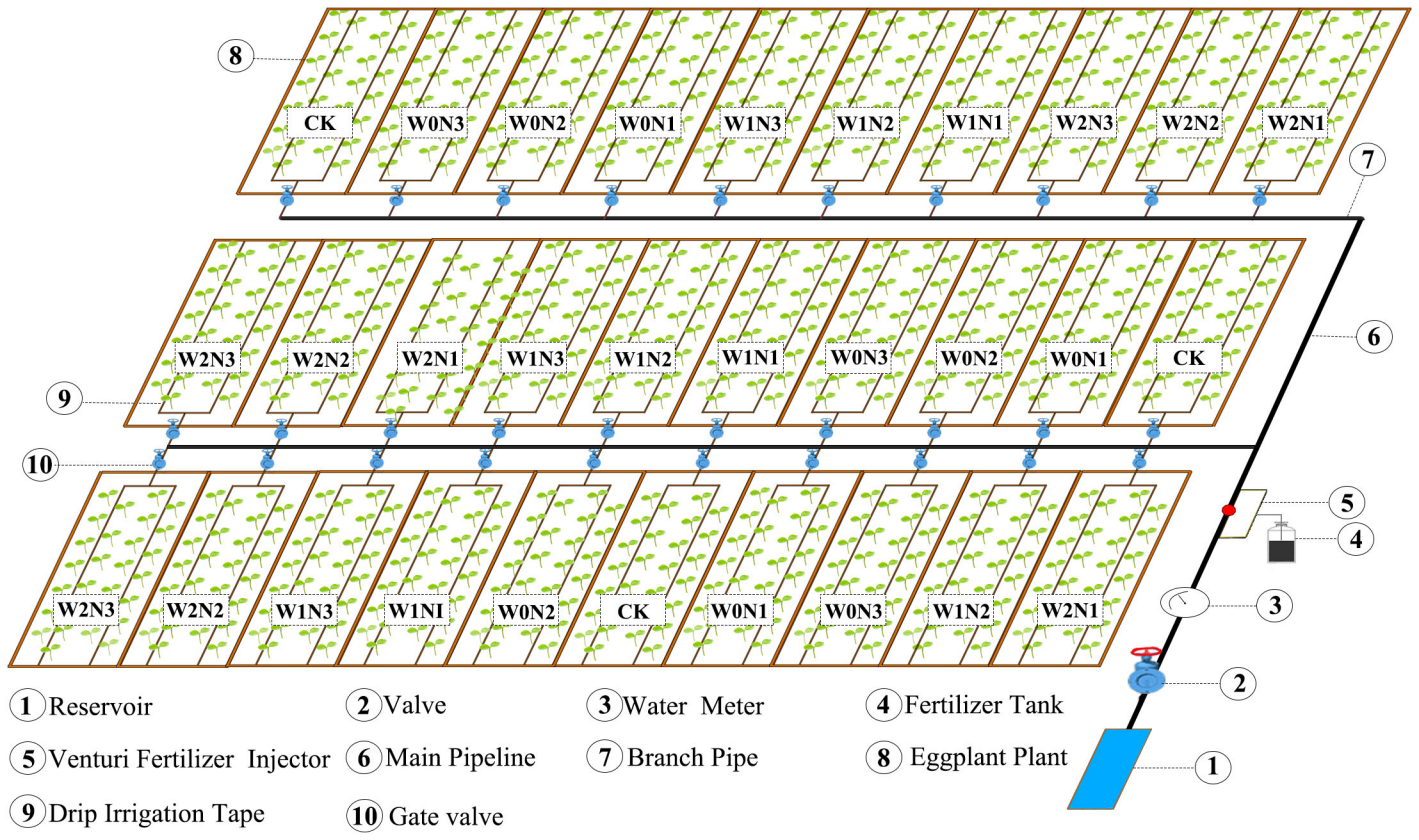
Schematic diagram of the experimental field layout.

**Figure 3:**
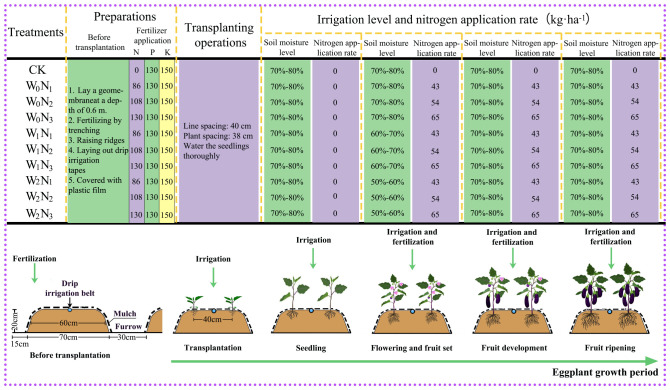
Irrigation and fertilization management, planting density, and sowing and harvesting dates for different treatments.

The present study utilized the Lan Za No. 2 eggplant variety and adopted the following cultivation method: single ridge with double rows and film mulching. Transplanting was completed on May 9 in 2021 and on May 11 in 2022. Each experimental plot was equipped with two ridges. The ridge body specifications were 20 cm in height, 600 cm in length, and 60 cm in width. A single-wing labyrinth drip irrigation tape was positioned centrally along the ridge and subsequently overlaid with a 120 cm wide plastic film. The transplanting configuration parameters were set at a row spacing of 40 cm and a plant spacing of 38 cm. The layout diagram for eggplant cultivation is shown in **Figure 2**. To prevent lateral seepage of water between test plots, a geomembrane with a burial depth of 60 cm was installed around each plot. Prior to transplanting the eggplant seedlings, all treatments received a uniform basal application of phosphorus fertilizer (superphosphate, 14% P_2_O_5_) and potassium fertilizer (potassium sulfate, 50% K_2_O), at application rates of 130 kg·ha^-1^ and 150 kg·ha^-1^, respectively. Urea (containing 46% nitrogen) was selected as the nitrogen fertilizer; 40% was applied as a basal fertilizer, and the remaining 60% was applied in three equal installments during the flowering and fruit set period, the fruit development period, and the fruit ripening period. Each application constituted 20% of the total nitrogen amount. Soil moisture was monitored at 5- to 7-day intervals throughout the experiment. Irrigation was triggered when moisture levels approached or dropped below the predetermined lower threshold, replenishing it to the established upper limit. The irrigation amounts applied at each growth stage and the total irrigation amount for the entire growth period under different treatments are presented in the **Table 2**. During the 2-year trial period, consistent agronomic management practices were implemented across all experimental treatments. This encompassed routine phytosanitary measures, including weed control and pest and disease management. Specific details regarding irrigation and fertilization management, planting density, and sowing and harvesting dates are provided in **Figure 3**.

**Table 2:** The irrigation amounts applied at each growth stage and the total irrigation amount for the entire growth period under different treatments.

Year	Treatments	Seedling/mm	Flowering and fruit set/mm	Fruit development/mm	Fruit ripening/mm	The entire growth period/mm
2021	CK	27.02	61.59	104.15	78.50	271.26
	W0N1	26.32	64.23	108.09	83.64	282.28
	W0N2	30.70	69.64	113.31	84.24	297.89
	W0N3	28.26	68.00	114.20	82.88	293.33
	W1N1	26.04	51.59	99.36	70.32	247.31
	W1N2	29.79	54.71	102.27	73.21	259.97
	W1N3	29.08	52.44	101.35	70.40	253.27
	W2N1	27.26	46.30	95.29	65.39	234.24
	W2N2	30.06	41.41	91.04	61.95	224.46
	W2N3	28.51	42.45	93.43	63.02	227.41
2022	CK	18.81	64.18	72.57	76.07	231.63
	W0N1	17.66	65.44	75.84	78.72	237.66
	W0N2	22.49	67.69	81.39	78.35	249.91
	W0N3	21.34	68.63	82.80	80.88	253.65
	W1N1	19.12	52.21	65.91	70.13	207.38
	W1N2	22.33	54.55	67.90	70.22	215.00
	W1N3	21.07	57.17	69.10	71.29	218.64
	W2N1	19.73	48.05	62.93	62.44	193.16
	W2N2	22.12	43.96	62.74	59.82	188.64
	W2N3	20.36	41.63	60.80	60.67	183.46

### 2.3 Measurements and calculations

#### 2.3.1 Meteorological parameters

**Figure 4:**
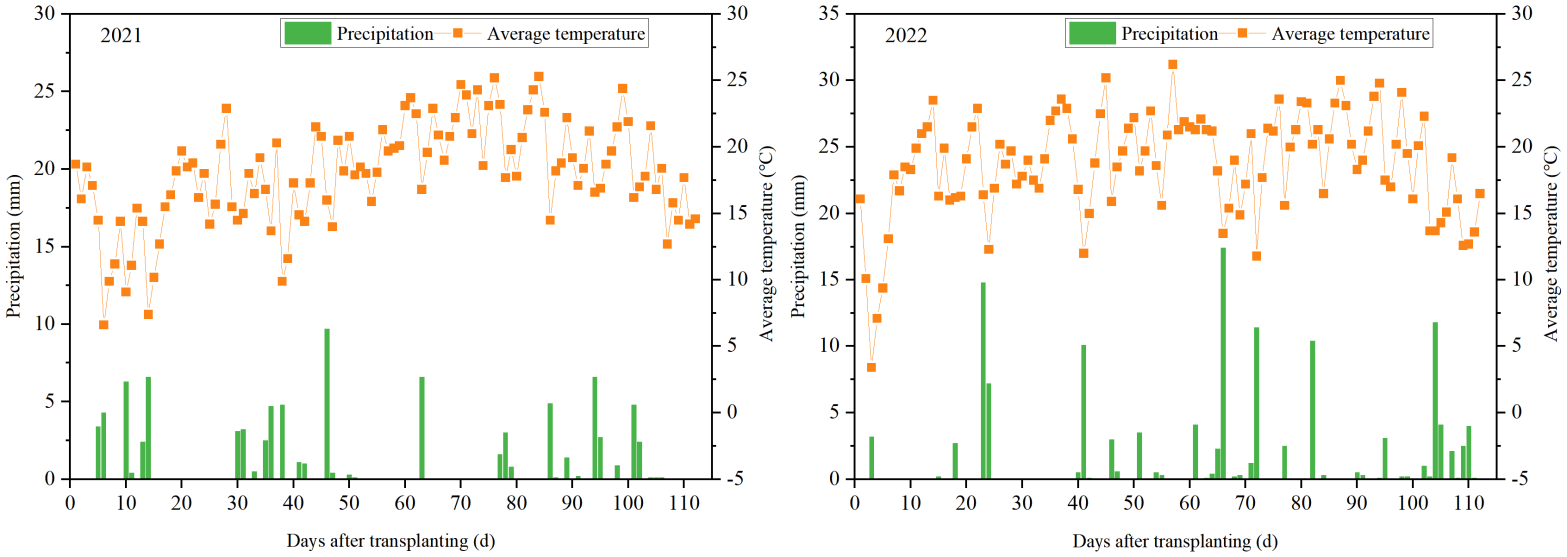
Precipitation and daily average temperature in the experimental areas in 2021 and 2022.

Meteorological parameters, including precipitation, atmospheric temperature, atmospheric relative humidity, sunshine duration, atmospheric pressure, and wind speed, were measured by the Minle County Meteorological Bureau at the Yimin Irrigation Experimental Station. The main meteorological parameters during the entire growth stage of eggplants for two consecutive years are provided in **Figure 4** and **Table 3**. The maximum temperatures for the first and second growing seasons were 33.2 °C and 35.1 °C, respectively, while the minimum temperatures were 2.7 °C and 0.6 °C, respectively. The mean temperatures for the growing seasons in 2021 and 2022 were 17.95 °C and 18.65 °C, respectively. For 2021 and 2022, the total precipitation amounts were 91.1 mm and 127.6 mm, respectively, while total effective precipitation amounts were 35.8 mm and 83.1 mm, respectively. The atmospheric relative humidity was 55.81% and 59.75% in 2021 and 2022, respectively. For the first and second growing seasons, the cumulative sunshine duration was 860.7 h and 802.8 h, respectively, while the average wind speeds were 1.52 m·s^-1^ and 1.47 m·s^-1^, respectively.

**Table 3:** Meteorological basic elements of the whole growth period of eggplant in 2021 and 2022.

Year	Growth period	Temperature/°C			Precipitation/mm	Effective rainfall/mm	Relative air humidity/%	Sunshine duration/h	Wind speed/m·s^-1^
		Maximum	Minimum	Mean					
2021	Seedling	27.2	2.7	14.37	23.4	12.9	43.68	158.9	1.75
	Flowering and fruit set	29.7	5.5	17.07	31.4	9.7	59.77	207.1	1.48
	Fruit development	33.2	9.3	21.26	16.9	6.6	55.37	283.7	1.46
	Fruit ripening	31.5	5.5	18.19	19.4	6.6	62.01	211	1.44
	Whole growth period	33.2	2.7	17.95	91.1	35.8	55.81	860.7	1.52
2022	Seedling	29.6	0.6	16.28	28.1	22.0	40.93	176	2.18
	Flowering and fruit set	32.2	7.8	19.09	18.6	10.1	51.21	266.1	1.41
	Fruit development	35.1	8.3	19.77	50.6	39.2	61.69	242.7	1.34
	Fruit ripening	32.0	9.3	18.71	30.3	11.8	79.47	118	1.15
	Whole growth period	35.1	0.6	18.65	127.6	83.1	59.75	802.8	1.47

The data were measured at the Yimin Irrigation Experimental station by the Minle Meteorological Bureau.

#### 2.3.2 Soil moisture

Soil water content was measured using the gravimetric method (Zhou et al., 2022). Soil moisture was determined once before the transplantation of eggplant seedlings, and subsequently every 5–7 days after transplantation, with additional measurements taken after rainfall and both before and after irrigation. The midpoint of the line connecting two adjacent eggplant seedlings on one side of the drip irrigation tape was randomly selected, and soil samples were collected layer−by−layer using a soil auger (at 20−cm intervals) to a depth of 100 cm. Since the root system of eggplant is mainly distributed in the 0–50 cm soil layer, the soil water content of the planned wetted layer was taken as the average of the soil water content in the 0–60 cm layer, and crop water consumption was calculated based on changes in soil water content in the 0–100 cm layer. The soil water content was calculated using the following formula:


$$\theta_{j}=\frac{m_{j1}-m_{j2}}{m_{j2}-m_{0}}\times 100\%$$

Where *θ_j_* is the soil water content of the j-th layer (%), *m_0_* is the mass of the aluminium box (g), *m_j1_* is the mass of the fresh soil plus aluminium box for the j-th layer (g), and *m_j2_* is the mass of the oven-dried soil plus aluminium box for the j-th layer (g).

The irrigation amount was calculated using the following formula (Zhou et al., 2022):


$$M=10\gamma HP(\theta_{2}-\theta_{1})$$

Where: *M* is the irrigation amount (mm); *γ* is the soil bulk density of the planned wetted layer (g·cm^-3^); *H* is the depth of the planned wetted layer (60 cm); *P* is the designed wetting ratio for drip irrigation; *θ_1_* is the measured soil water content (%); and *θ_2_* is the upper limit of soil water content as specified in the experimental design (%).

#### 2.3.3 Growth indicators

For measurement of plant height (PH), stem diameter (SD), and leaf area, five eggplant plants with normal and uniform growth were selected in each experimental plot, marked, and then monitored throughout their entire growth period. Eggplant PH was determined with a steel tape measure (accurate to 0.1 cm), while SD was measured using a vernier caliper (precision of 0.02 mm). Leaf area was assessed by means of a leaf area meter. Leaf area index (LAI) was derived by multiplying the leaf area per plant by the plant density per unit area.

For dry matter measurement, sampling was conducted at the end of each reproductive stage. Three evenly grown and normally developing eggplant plants were dug out from each experimental plot, promptly transported to the lab for rinsing, and then blotted with filter paper to remove excess water. The roots, stems, leaves, and fruits (from both fruit development and fruit ripening stages) were separated using scissors. After quickly weighing and recording their fresh weights, each part was placed into a pre-labeled paper bag. Following a deactivation step in an oven at 105 °C for 30 minutes, the samples were dried at 85 °C until a constant weight was attained. Subsequently, the dry weights of the component were measured and recorded (Wu et al., 2023). The calculation of dry matter allocation percentages is the ratio of the dry matter mass of that part to the total dry matter accumulation of the eggplant plant.

#### 2.3.4 Yield

The eggplant yield was harvested in four batches according to maturity stages. After the fruits ripened in each cropping cycle, the experimental plots were harvested independently. To ensure representativeness, the yield data for each treatment were derived from the mean value obtained across three experimental replicates.

#### 2.3.5 Water and nitrogen use efficiencies

Crop water productivity (*WP_C_*; kg·m^−3^) was determined according to the following formula (Fernández et al., 2020):


$$WP_{C}=\frac{Y}{ET_{C}}$$

Irrigation water productivity (*WP_I_*; kg·m^−3^) was derived from the following formula (Fernández et al., 2020):


$$WP_{I}=\frac{Y}{I}$$

Nitrogen partial factor productivity (*NPFP*; kg·kg^−1^) was determined as follows (Thompson, 2023):


$$\;NPFP=\frac{Y}{NFR}$$

In the equations, *Y* corresponds to the total yield of eggplant (t·ha^−1^), *ET_C_* indicates the total water consumption throughout the entire growth period (m^3^·ha^−1^), *I* denotes the total irrigation amount over the growth period (m^3^·ha^−1^), *NFR* indicates the nitrogen fertilizer application rate (kg·ha^−1^).

#### 2.3.6 Comprehensive evaluation method for optimal irrigation and nitrogen fertilization schemes for eggplant

(1) Construction of the technique for order preference by similarity to ideal solution

The technique for order preference by similarity to ideal solution (TOPSIS) method involves the following calculation steps:

1) construct the original decision matrix


$$X={\left(x_{ij}\right)}_{m\times n}=[\begin{array}{ccc} \begin{array}{cc} x_{11} & x_{12}\\ x_{21} & x_{22} \end{array} & \begin{array}{c} \ldots \\ \ldots \end{array} & \begin{array}{c} x_{1n}\\ x_{2n} \end{array}\\ \begin{array}{cc} \vdots & \vdots \end{array} & \ddots & \vdots \\ \begin{array}{cc} x_{m1} & x_{m2} \end{array} & \cdots & x_{mn} \end{array}]$$

Where *x_ij_* (*i* = 1, 2…, *m*; *j* = 1, 2…, *n*) is the j-th evaluation objective of the i-th treatment.

2) Original data and trend normalization (positive transformation of indicators)

For extremely small indicators:


$$\;x^{\prime }=M-x$$

3) Normalize the original matrix to construct a standardized matrix


$$Z_{ij}=W_{j}\frac{x_{ij}}{\sqrt{\sum_{i=1}^{m}x_{ij}^{2}}}$$

Where *Z_ij_* represents the standardization of *x_ij_*; *W_j_* is the weight of the jth evaluation indicator. In this study, *W_j_* is set to 1.

4) Determine the optimal solution *Z^+^* and the worst solution *Z^-^*


$$Z^{+}=(Z_{max\;1},Z_{max\;2,}\cdots ,Z_{max\;n})$$

$$Z^{-}=(Z_{min\;1},Z_{min\;2,}\cdots ,Z_{min\;n})$$

5) Calculate the distances *D_i_*^+^ and *D_i_*^−^ from each evaluation object to *Z*^+^ and *Z*^−^


$$D_{i}^{+}=\sqrt{\overset{n}{\underset{j=1}{\sum }}{\left(Z_{ij}-Z_{j}^{+}\right)}^{2}}$$

$$D_{i}^{-}=\sqrt{\overset{n}{\underset{j=1}{\sum }}{\left(Z_{ij}-Z_{j}^{-}\right)}^{2}}$$

6) Calculate the degree of closeness of each evaluation object to the optimal solution


$$C_{i}=\frac{D_{i}^{-}}{D_{i}^{+}+D_{i}^{-}}$$

(2) Construction of the grey relational analysis

1) Construct the original decision matrix


$$A={\left(a_{ij}\right)}_{m\times n}=[\begin{array}{ccc} \begin{array}{cc} a_{11} & a_{12}\\ a_{21} & a_{22} \end{array} & \begin{array}{c} \ldots \\ \ldots \end{array} & \begin{array}{c} a_{1n}\\ a_{2n} \end{array}\\ \begin{array}{cc} \vdots & \vdots \end{array} & \ddots & \vdots \\ \begin{array}{cc} a_{m1} & a_{m2} \end{array} & \cdots & a_{mn} \end{array}]$$

Where *a_ij_* (*i* = 1, 2…, *m*; *j* = 1, 2…, *n*) is the j-th evaluation objective of the i-th treatment.

2) Normalization of raw data


$$X_{ij}=\frac{a_{ij}}{\bar{a_{ij}}}$$

Where *X_ij_* represents the standardization of *a_ij_*, where *a_ij_* is the j-th evaluation target of the i-th treatment, and $\bar{a_{ij}}$ is the mean value of all evaluation indicators.

3) Determine the reference sequence


$$X_{0}=(x_{max\;1},\;x_{max\;2},\;\cdots ,\;x_{max\;n})$$

Where *x_max j_* represents the maximum value of the n-th evaluation indicator among all treatments, and j=1, 2…, n.

4) Calculate the correlation coefficient matrix


$$\epsilon_{ik}=\frac{\begin{array}{c} min\\ i \end{array}\begin{array}{c} min\\ k \end{array}|x_{0k}-x_{ik}|+\rho \begin{array}{c} max\\ i \end{array}\begin{array}{c} max\\ k \end{array}|x_{0k}-x_{ik}|}{\left|x_{0k}-x_{ik}|+\rho \begin{array}{c} max\\ i \end{array}\begin{array}{c} max\\ k \end{array}|x_{0k}-x_{ik}\right|}$$

Where *ρ* represents the resolution coefficient, and its value range is (0, 1). In this study, *ρ=*0.5.

5) Calculate the correlation degree *γ_i_*


$$\gamma_{i}=\frac{\sum_{k=1}^{n}\epsilon_{ik}}{n}$$

### 2.4 Data processing and statistical analysis

All experimental data are expressed as the mean values derived from three independent replicates. Given that the two-year trial was conducted under different climatic conditions, all statistical analyses were performed separately for each year to avoid confounding inter-annual variations with treatment effects. A two-way analysis of variance (ANOVA) was performed using IBM SPSS Statistics 23.0 to evaluate the main effects of irrigation, nitrogen rate, and their interaction on the measured parameters for each year independently. Mean comparisons among treatments were conducted using Duncan’s multiple range test, with statistical significance set at P ≤ 0.05. Pearson correlation analyses were also performed separately for each year’s dataset to examine the relationships between key variables. Graphical representations of the data were generated using Origin 2021. Furthermore, a comprehensive evaluation was conducted based on 12 indicators related to agronomic traits, yield, and water–nitrogen use efficiency. This assessment employed the technique for order preference by similarity to ideal solution (TOPSIS) in conjunction with grey relational analysis (GRA).

## 3 Results

### 3.1 Plant growth

#### 3.1.1 Plant height

**Figure 5:**
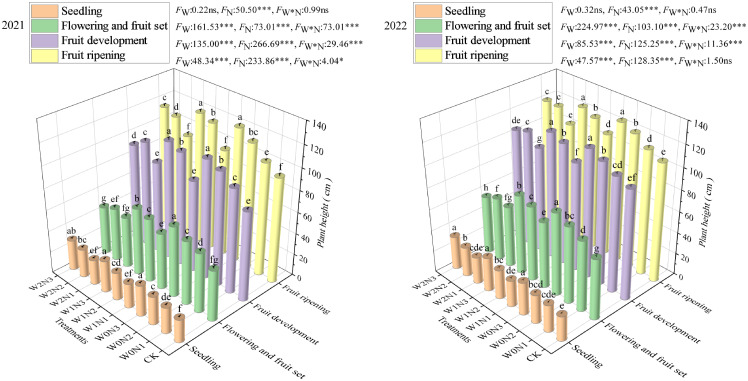
Effects of different water and nitrogen treatments on eggplant plant height. Different lowercase letters within a column indicate statistically significant differences among treatments (*P* < 0.05). *F_W_* represents the F–value of the split-plot ANOVA for irrigation, *F_N_* represents the F–value for nitrogen application, and *F_W×N_* represents the F–value for the interaction between irrigation and nitrogen application. *P < 0.05, **P < 0.01, ***P < 0.001, ns indicates not statistically significant.

During the seedling stage, nitrogen application exerted a highly significant (*P <* 0.001) effect on plant height (PH). Plants treated with W1N3 exhibited the tallest height (**Figure 5**), with no significant differences compared with the W0N3 and W2N3 treatments (*P* > 0.05). During the flowering and fruit set period, plants treated with W0N3 exhibited the tallest height. Compared with that of the W1N3 treatment, the W0N3 treatment significantly increased (*P* < 0.05) the PH by 4.83% (2021) and 4.51% (2022), respectively. Relative to the CK treatment, the W0N3 treatment significantly increased the PH by 39.71% (2021) and 37.02% (2022), respectively. The mean PH was maximized under the W1 level, which was similar to that under the W0 level but significantly higher than that under the W2 level by 22.33% (2021) and 19.08% (2022), respectively. Under consistent nitrogen application rate, the mean PH increased with increasing nitrogen application levels. The N3 treatment resulted in the tallest plants, showing no significant difference compared with those under the N2 treatment but significantly exceeded the PH of those under the N1 treatment by 11.59% in 2021 and 11.21% in 2022. At the fruit development stage, the PH was greatest under the W0N3 treatment, and it was at the same level as that under the W1N3 treatment. During the fruit ripening period, the tallest PH was obtained under the W0N3 treatment (128.90 cm), showing no significant difference from that under the W1N3 treatment. The shortest PH was obtained under the W2N1 treatment, and the height was comparable to that obtained under the CK treatment. At the W0 and W1 levels, the PH increased with the increase in nitrogen application rate. However, at the W2 level, the PH increased initially with the increase in nitrogen application rate but subsequently decreased.

#### 3.1.2 Stem diameter

**Figure 6:**
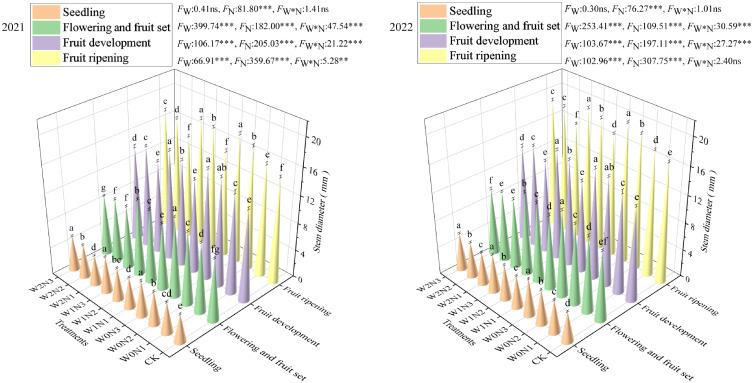
Effects of different water and nitrogen treatments on eggplant stem diameter. Different lowercase letters within a column indicate statistically significant differences among treatments (*P* < 0.05). *F_W_* represents the F–value of the split-plot ANOVA for irrigation, *F_N_* represents the F–value for nitrogen application, and *F_W×N_* represents the F–value for the interaction between irrigation and nitrogen application. *P < 0.05, **P < 0.01, ***P < 0.001, ns indicates not statistically significant.

Irrigation and nitrogen application exerted highly significant (*P* < 0.001) effects on eggplant stem diameter (SD) across the flowering and fruit set, fruit development, and fruit ripening periods (**Figure 6**). The irrigation and nitrogen application interaction was significant (*P* < 0.001) only during the flowering and fruit set and fruit development periods. During the seedling stage, the SDs ranged from 4.06 to 5.23 mm (2021) and from 4.37 to 5.51 mm (2022). The SD under the CK treatment was the lowest, with significant (*P* < 0.05) decreases of 6.45–22.37% (2021) and 6.02–20.69% (2022) compared with the other treatments. During flowering and fruit set period, the SD increased with increasing nitrogen application rates at the W0 and W1 levels. At the W2 level, the SD was greatest at the N2 level, showing no significant difference compared with that at the N1 level. Moreover, the SD at the N3 level was significantly reduced by 4.74% (2021) and 4.85% (2022) compared with that at the N2 level. At the N1 and N3 levels, the SD exhibited a progressive reduction with increasing severity of water deficit. During the fruit development stage, the SD under the W0N3 treatment was the greatest but had no statistically significant difference compared with that under the W1N3 treatment. At the W2 level, the SD exhibited an initial increase, which was followed by a decrease as the nitrogen application rate rose. However, at W0 and W1 levels, the SD consistently increased with higher nitrogen application rates. At the fruit ripening stage, the SDs ranged from 15.17 to 18.65 mm (2021) and from 16.89 to 20.10 mm (2022). The greatest SD occurred at the W0N3 level, showing significant increases of 21.82% (2021) and 15.98% (2022) compared with that under the CK treatment.

#### 3.1.3 Leaf area index

**Figure 7:**
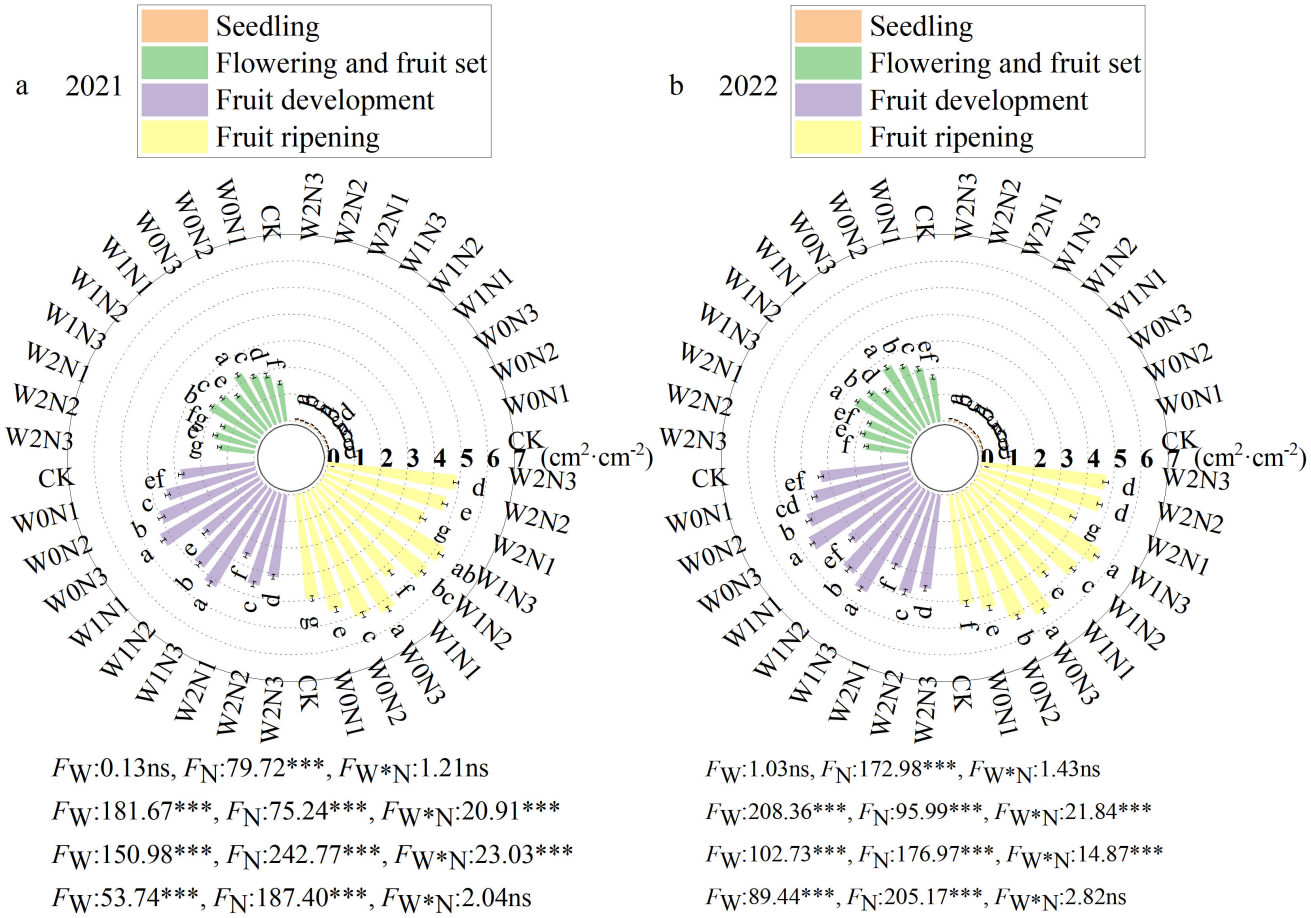
Effects of different water and nitrogen treatments on eggplant leaf area index. Different lowercase letters within a column indicate statistically significant differences among treatments (*P* < 0.05). *F_W_* represents the F–value of the split-plot ANOVA for irrigation, *F_N_* represents the F–value for nitrogen application, and *F_W×N_* represents the F–value for the interaction between irrigation and nitrogen application. *P < 0.05, **P < 0.01, ***P < 0.001, ns indicates not statistically significant.

Irrigation and nitrogen application significantly (*P* < 0.001) influenced the leaf area index (LAI), except during the seedling period (**Figure 7**). The irrigation and nitrogen application interaction was significant (P < 0.001) only during the flowering and fruit set and fruit development periods. During the seedling stage, the mean LAI values across all nitrogen application rates significantly increased with increasing nitrogen levels (*P* < 0.05), and the lowest LAI occurred under the CK treatment. The highest eggplant LAI mean value was observed at the N3 level; compared with those at the N1 and N2 levels, the LAI values at the N3 level significantly increased by 43.10% and 20.29% in 2021 (*P* < 0.05), as well as by 50.00% and 18.63% in 2022, respectively. During the flowering and fruit set period, the LAI under the W0N3 treatment in 2021 was the highest, significantly exceeding other water–nitrogen treatments by 6.65–74.45%. In 2022, the LAI under the W0N3 treatment was the highest, showing no significant difference from that under the W1N3 treatment and significantly exceeding other water–nitrogen treatments by 10.88–66.46%. At the N2 level, the LAI exhibited an initial increase, which was followed by a decrease with intensifying water deficit. At the N1 and N3 levels, the LAI decreased as the water deficit intensified. During the fruit development period, owing to the compensatory growth effect on plants after rehydration, the LAI under the W1N3 treatment in both growing seasons was at the same level as that under the W0N3 treatment but significantly higher than that of other treatments. At the fruit ripening period, the lowest LAI in 2021 occurred under the CK treatment, and the lowest LAI in 2022 occurred under the W2N1 treatment, with a significant decrease of 5.25% compared with that under the CK treatment.

### 3.2 Dry matter accumulation and dry matter distribution

#### 3.2.1 Dry matter accumulation

**Figure 8:**
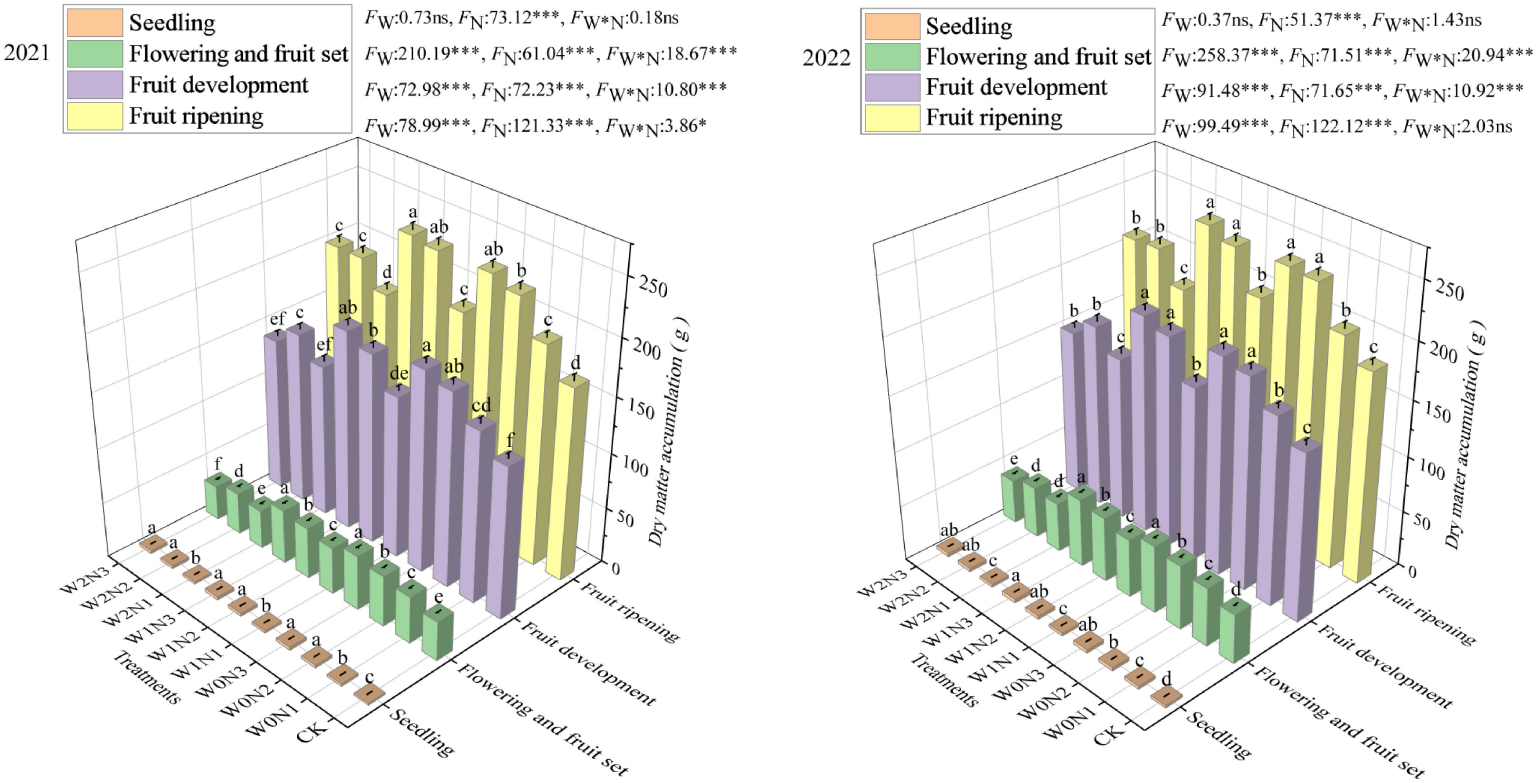
Effects of different water and nitrogen treatments on eggplant dry matter accumulation. Different lowercase letters within a column indicate statistically significant differences among treatments (*P* < 0.05). *F_W_* represents the F–value of the split-plot ANOVA for irrigation, *F_N_* represents the F–value for nitrogen application, and *F_W×N_* represents the F–value for the interaction between irrigation and nitrogen application. *P < 0.05, **P < 0.01, ***P < 0.001, ns indicates not statistically significant.

Total dry matter accumulation (DMA) per plant increased progressively throughout the growth cycle, peaking at fruit ripening (**Figure 8**), following a characteristic sigmoidal (S-shaped) growth curve. Growth was slow during the seedling and fruit ripening stages but vigorous throughout the flowering and fruit set stage and the fruit development stage. Both irrigation and nitrogen application exerted highly significant (*P* < 0.001) effects on total DMA across the entire growth period. At the seedling stage, the CK treatment exhibited the lowest DMA, which was significantly lower (*P* < 0.05) than that under other treatments by 18.78–39.51% (2021) and 12.69–34.20% (2022). During the flowering and fruit set stage, no statistically significant difference (*P* > 0.05) was found between the W0N3 and W1N3 treatments. In 2021, the highest DMA was recorded under the W0N3 treatment, exceeding other treatments by 6.76–42.07%. In 2022, the highest DMA was obtained under the W1N3 treatment, surpassing others by 5.50–55.02%. In 2021, the average DMA of eggplant under the W2 treatment decreased significantly by 19.80% and 24.35% compared with that under the W0 and W1 treatments, respectively. In 2022, the highest average DMA was recorded under the W1 treatment, which increased significantly by 6.68% and 32.63% compared with that under the W0 and W2 treatments, respectively. The lowest DMA occurred under the W2N3 treatment, showing a significant 10.10% reduction compared with that under the CK treatment. During fruit development, the DMA increased significantly compared to the previous stage. Among the treatments, the W0N2, W0N3, W1N2, and W1N3 treatments showed similar DMA levels, with significantly higher DMA levels compared with those under the W2N2 and W2N3 treatments. These findings indicated that rehydration after mild water deficit enhances DMA, whereas compensation is limited following moderate water deficit. At the fruit ripening stage, the average DMA at the N2 and N3 levels remained comparable and was significantly higher than that at the N1 level by 16.69–18.64% (2021) and 15.53–17.07% (2022).

#### 3.2.2 Dry matter distribution

**Figure 9:**
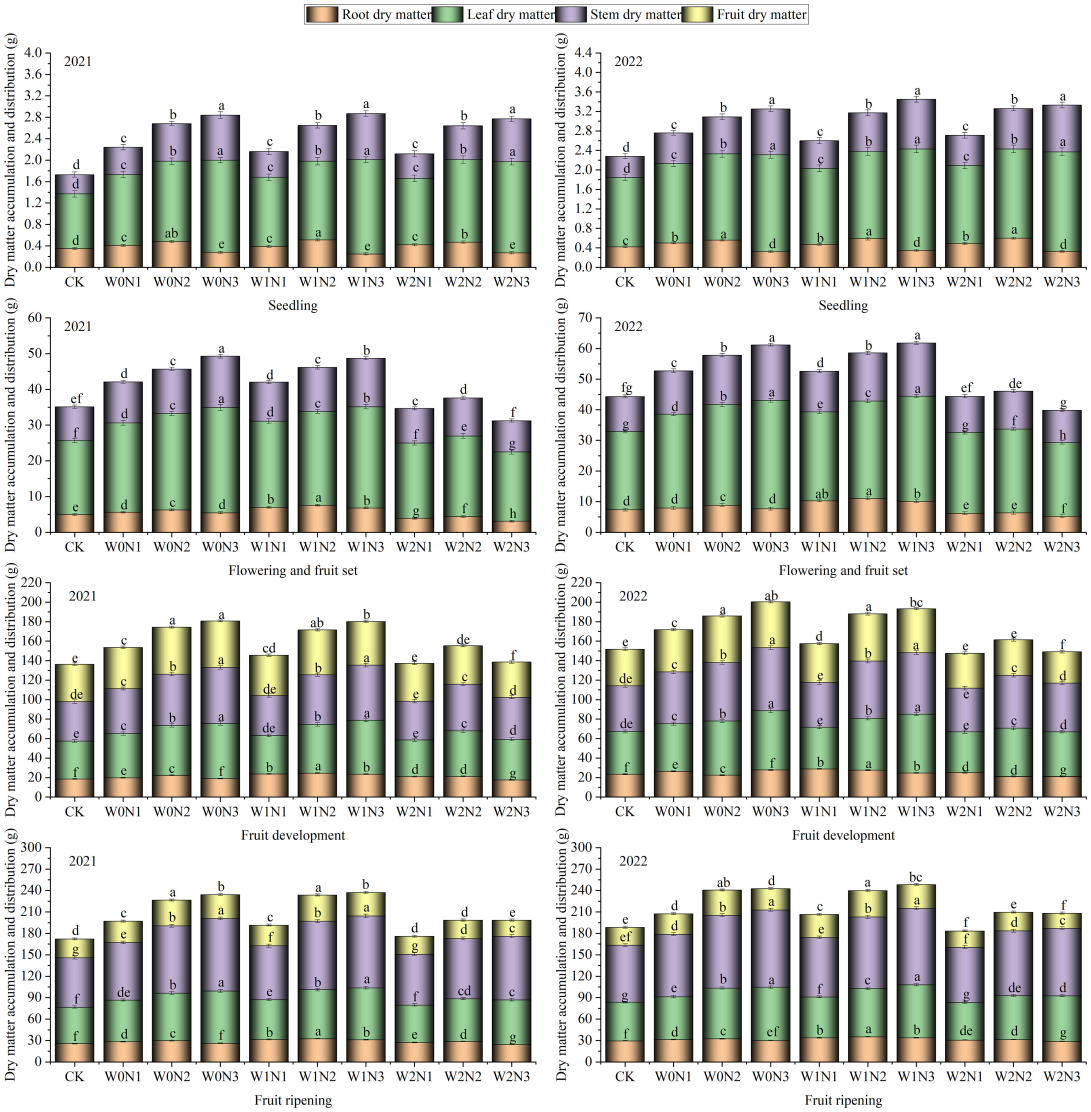
Effects of different water and nitrogen treatments on dry matter distribution of eggplant. Different lowercase letters within a column indicate statistically significant differences among treatments (*P* < 0.05).

During reproductive development, the DMA in leaves, stems, and roots of eggplant continuously increased, peaking at the end of the fruit ripening stage (**Figure 9**). The accumulation rate was highest in leaves from the seedling stage to the flowering and fruit set stage, while the accumulation rate was highest in stems and roots from the flowering and fruit set state to the fruit development stage. At the seedling stage, leaves constituted the largest proportion of DMA, followed by stems, with roots constituting the smallest proportion. Upon entering the flowering and fruit set stage, vigorous vegetative growth favored aboveground parts, increasing DMA in leaves (57.45–60.46% in 2021 and 54.43–60.46% in 2022) and stems (25.95–29.18% in 2021 and 25.14–29.63% in 2022) while decreasing DMA in roots (9.94–16.61% in 2021 and 12.60–19.57% in 2022). During fruit development, concurrent vegetative and reproductive growth led to a gradual increase in fruit DMA, reaching 24.87–28.49% (2021) and 21.57–25.62% (2022) by the end of this stage. In the fruit ripening stage, stem growth surpassed leaf growth. The proportion of fruit DMA decreased compared with those in earlier stages, with proportion of fruit DMA ranging from 11.38% to 15.87% in 2021 and from 10.10% to 15.56% in 2022. During the fruit ripening period, the root DMA increased, followed by a decrease with increasing nitrogen rates, peaking at the N2 level. Similarly, the fruit DMA was highest at the N2 level, showing significant increases of 17.33% (2021) and 17.61% (2022) compared with that at the N1 level, as well as significant increases of 11.14% (2021) and 16.52% (2022) compared with that at the N3 level (*P* < 0.05).

### 3.3 Yield and yield components

#### 3.3.1 Plant yield

Irrigation, nitrogen application, and their interaction during the two growing seasons all exerted significant (*P* < 0.001) effects on yield (**Table 4**). The W1N2 treatment maintained the highest total yield, which was comparable to that of the W0N2 treatment but significantly exceeded the yields of other water–nitrogen treatments by 11.14–48.99% (2021) and 10.78–49.41% (2022). Under the W0 and W1 levels, the yields at the N2 level were significantly greater than those at the N1 and N3 levels. Under the W2 level, the N2 level produced the highest yield, showing no significant difference compared with that at the N1 level (*P* > 0.05) but significant increases of 11.91% (2021) and 12.48% (2022) compared with that at the N3 level (*P* < 0.05). When evaluated at consistent irrigation levels, the highest average yield was observed under the W1 treatment. Compared with that at the W0 level, the average yield at the W1 level increased significantly (*P* < 0.05) by 3.66% in 2021 and by 4.49% in 2022. The average yield reached its maximum at the N2 level, and the yield at the N3 level was significantly (*P* < 0.05) lower than that at the N2 level by 10.65% (2021) and 10.12% (2022).

**Table 4:** Effects of different water and nitrogen treatments on yield and yield components of eggplant.

Treatments	2021			2022		
	Yield (t·ha^-1^)	Fruit number per plant	Fruit weight (g)	Yield (t·ha^-1^)	Fruit number per plant	Fruit weight (g)
CK	64.07 ± 1.77d	6.37 ± 0.32c	200.61 ± 3.71d	65.06 ± 1.92d	6.37 ± 0.21d	199.94 ± 4.50d
W0N1	70.35 ± 1.27c	7.03 ± 0.25b	215.68 ± 3.85c	72.74 ± 2.52c	6.93 ± 0.15bc	220.43 ± 4.38c
W0N2	84.15 ± 0.18a	8.10 ± 0.30a	243.77 ± 3.68a	85.60 ± 1.67a	7.97 ± 0.35a	249.69 ± 2.98a
W0N3	76.45 ± 2.21b	7.70 ± 0.30a	232.76 ± 3.47b	79.32 ± 2.87b	7.57 ± 0.21a	236.47 ± 5.14b
W1N1	69.75 ± 0.79c	7.07 ± 0.32b	211.01 ± 5.33c	72.12 ± 1.80c	6.97 ± 0.31b	218.29 ± 4.38c
W1N2	84.97 ± 1.30a	8.00 ± 0.36a	247.08 ± 3.26a	87.87 ± 1.97a	7.87 ± 0.25a	252.17 ± 3.77a
W1N3	74.65 ± 1.37b	7.90 ± 0.26a	230.00 ± 4.50b	77.23 ± 1.44b	7.73 ± 0.25a	233.41 ± 3.85b
W2N1	62.79 ± 1.33d	6.23 ± 0.38c	195.71 ± 3.79d	63.57 ± 1.49d	6.30 ± 0.26d	196.76 ± 4.15d
W2N2	63.82 ± 1.84d	6.30 ± 0.36c	198.31 ± 5.13d	66.15 ± 2.48d	6.50 ± 0.17cd	201.58 ± 3.81d
W2N3	57.03 ± 1.67e	6.07 ± 0.32c	184.76 ± 4.50e	58.81 ± 2.25e	6.03 ± 0.35d	183.17 ± 4.67e
FW	332.02***	60.08***	237.26***	182.79***	66.87***	288.88***
FN	136.42***	18.44***	82.47***	75.43***	25.18***	100.73***
FW*N	24.60***	2.94*	17.09***	10.44***	4.05*	15.21***

Different lowercase letters indicate statistically significant differences between treatments (*p* < 0.05). *F_W_* represents the F–value of the split-plot ANOVA for irrigation, *F_N_* represents the F–value for nitrogen application, and *F_W×N_* represents the F–value for the interaction between irrigation and nitrogen application. *P < 0.05, **P < 0.01, ***P < 0.001.

#### 3.3.2 Yield components

Irrigation and nitrogen application exerted highly significant (*P* < 0.001) effects on the fruit number per plant (FN), while their interaction showed a significant influence (*P* < 0.05) on FN (**Table 4**). Furthermore, the single fruit weight (FW) was significantly affected by irrigation, nitrogen application, and their interaction (*P* < 0.001). The FN under different treatments ranged from 6.03 to 8.10 across the two growing seasons. The FN under the W0N2 treatment was comparable to that under the W0N3, W1N2, and W1N3 treatments. Compared with the CK, it was significantly increased (*P* < 0.05) by 27.16% (2021) and 25.12% (2022), respectively. Relative to the remaining water−nitrogen treatments, it was significantly (P < 0.05) elevated by 14.57–33.44% in 2021 and 14.35–32.17% in 2022, respectively. At the same irrigation level, the FN exhibited an initial increase, followed by a decrease with increasing nitrogen application rates. Under the same nitrogen application level, the FN at the W1 level was comparable to that at the W0 level (*P* > 0.05). The FN at the W2 level was significantly lower than both mild water deficit and adequate water supply. The W1N2 yielded the highest FW, which was comparable to the FW under the W0N2 treatment but significantly exceeded the FW of other water–nitrogen treatments by 6.15–33.73% (2021) and 6.64–37.67% (2022). At a given irrigation level, the FW exhibited an initial increase, followed by a decrease with increasing nitrogen application rates. Under consistent nitrogen application rate, the FW at the W1 level was comparable to that at the W0 level. In 2021, the FW at the W2 level was significantly reduced by 7.25–19.74% compared with that at the W1 level. In 2022, the FW was significantly reduced by 9.86–21.52% compared with that at the W1 level.

### 3.4 Water and nitrogen fertilizer use efficiencies

#### 3.4.1 Crop water productivity

**Figure 10:**
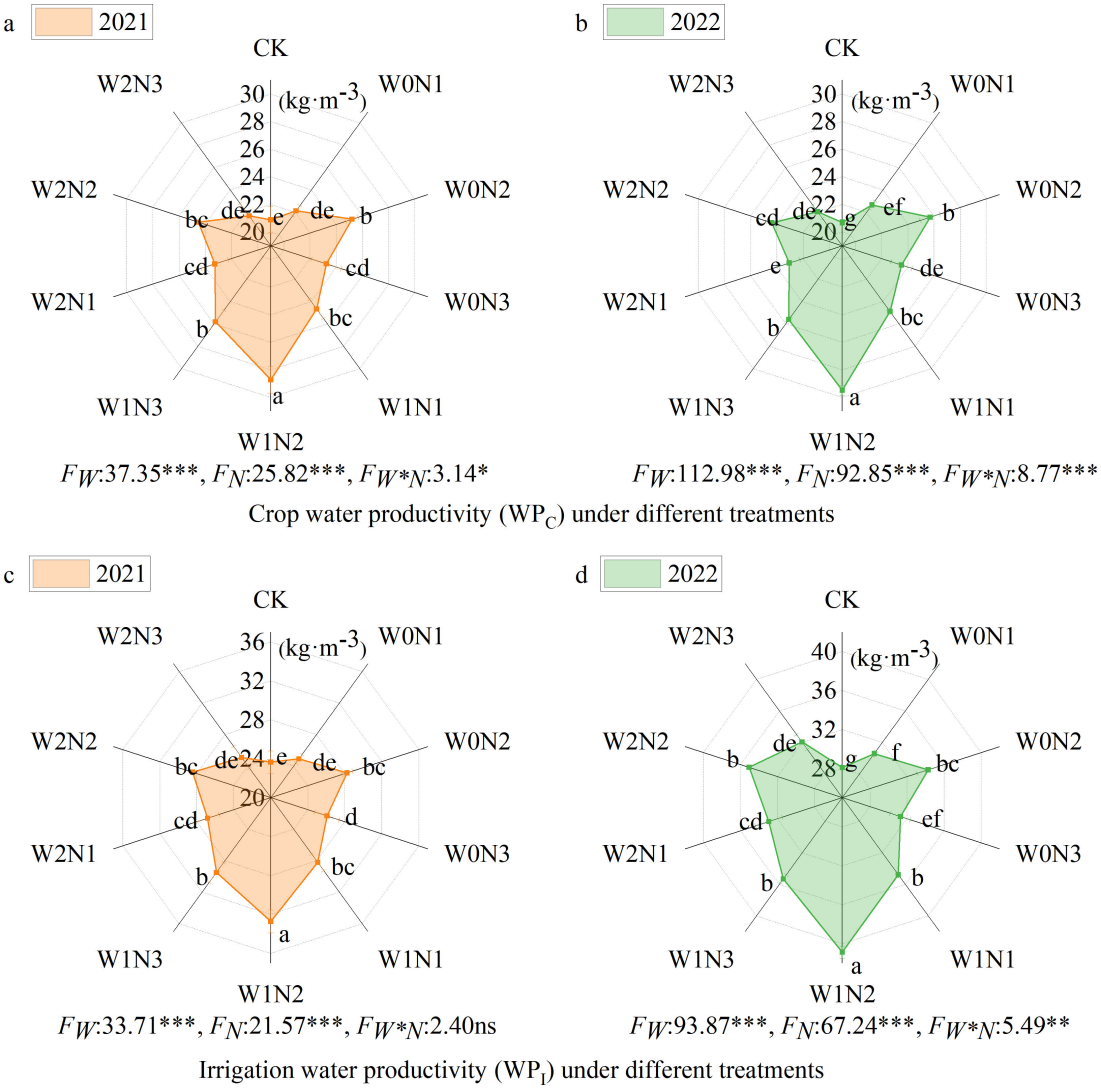
Effects of different water and nitrogen treatments on crop water productivity (*WP_C_*) and irrigation water productivity (*WP_I_*). Different lowercase letters within a column indicate statistically significant differences among treatments (*P* < 0.05). *F_W_* represents the F–value of the split-plot ANOVA for irrigation, *F_N_* represents the F–value for nitrogen application, and *F_W×N_* represents the F–value for the interaction between irrigation and nitrogen application. *P < 0.05, **P < 0.01, ***P < 0.001, ns indicates not statistically significant.

The effects of irrigation and nitrogen application on crop water productivity (*WP_C_*) and irrigation water use productivity (*WP_I_*) in eggplant were highly significant (*P* < 0.001) (**Figure 10**). The highest *WP_C_* was recorded under the W1N2 treatment, showing significant increases (*P* < 0.05) of 11.27–37.60% (2021) and 14.71–42.48% (2022) compared with that of other treatments. Under consistent irrigation conditions, the *WP_C_* value first increased and then decreased as the nitrogen application rate increased. Regarding the average *WP_C_* under the same irrigation levels, the *WP_C_* exhibited a comparable pattern in response to progressive water deficit, increasing to an optimum before decreasing. Compared with sufficient water supply, the *WP_C_* at the W1 level exhibited significant increases of 15.53% in 2021 and 15.17% in 2022. Under a constant nitrogen application rate, the *WP_C_* exhibited an initial increase, followed by a decrease as water deficit became more severe. When comparing the average *WP_C_* across the nitrogen levels, the N2 treatment consistently outperformed the N1 treatment (with increases of 11.94% in 2021 and 12.71% in 2022) and the N3 treatment (with increases of 10.90% in 2021 and 11.76% in 2022). The W1N2 treatment yielded the highest *WP_I_*, showing significant increases of 38.33% (2021) and 45.53% (2022) compared with that under the CK treatment. The W1N2 treatment also significantly outperformed other the treatments by 10.85–31.16% in 2021 and by 15.71–33.60% in 2022. Under consistent irrigation conditions, the WP_I_ first increased and then decreased as nitrogen application rates increased. Similarly, when the nitrogen application was consistent, the *WP_I_* first increased and then decreased as the water deficit increased. The *WP_I_* at the W1 level was the highest, showing significant increases of 13.11–15.71% compared with that at the W0 level in 2021, as well as significant increases of 12.95–19.29% compared with that at the W0 level in 2022.

#### 3.4.2 Nitrogen fertilizer use efficiency

**Figure 11:**
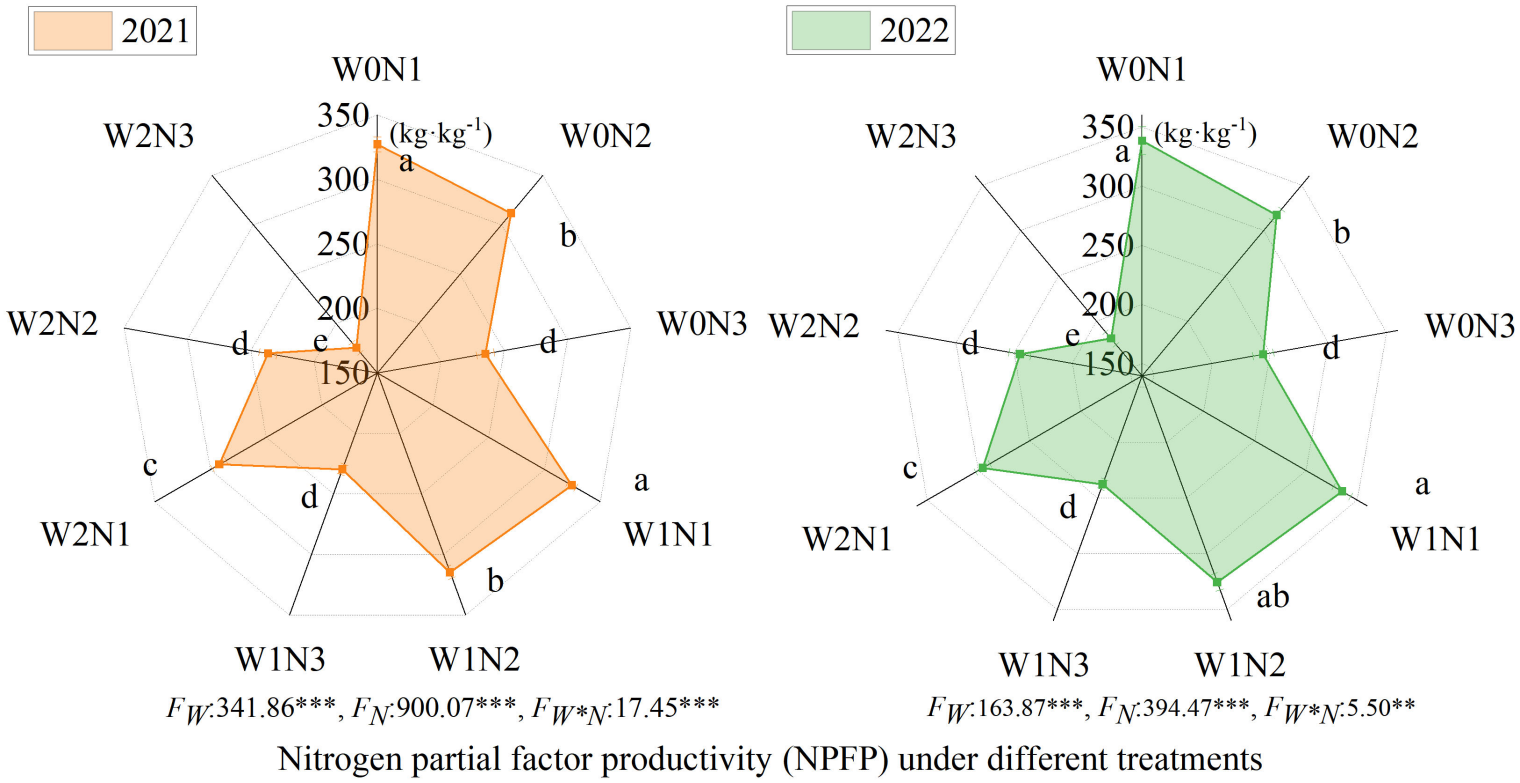
Effects of different water and nitrogen treatments on nitrogen partial factor productivity (NPFP). Different lowercase letters within a column indicate statistically significant differences among treatments (*P* < 0.05). *F_W_* represents the F–value of the split-plot ANOVA for irrigation, *F_N_* represents the F–value for nitrogen application, and *F_W×N_* represents the F–value for the interaction between irrigation and nitrogen application. *P < 0.05, **P < 0.01, ***P < 0.001.

The effects of irrigation, nitrogen application, and their interaction on nitrogen partial factor productivity (NPFP) was all statistically significant (P<0.001 or P<0.01) (**Figure 11**). The NPFP under the W2N3 treatment showed the lowest values, which were significantly reduced (*P* < 0.05) by 23.60–46.37% in 2021 and by 23.86–46.52% in 2022 compared with the other treatments. Under consistent irrigation conditions, the NPFP exhibited a significant decrease as the nitrogen application rates increased. Specifically, the NPFP at the N1 level was markedly higher compared with that at the N2 and N3 levels. At the N1 and N2 levels, no statistically significant difference in NPFP values was observed between the W0 and W1 treatments.

### 3.5 Pearson correlation analysis

**Figure 12:**
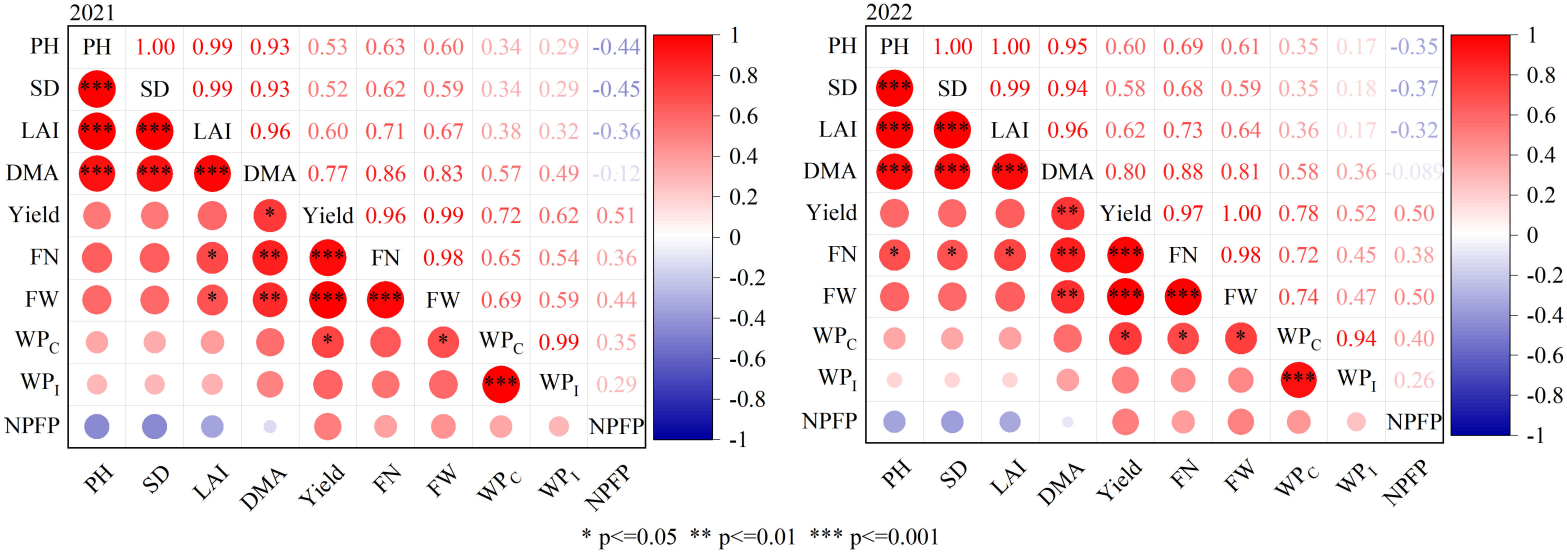
Correlation analysis between indicators in 2021 **(A)** and 2022 **(B)**. PH: plant height; SD: stem diameter; LAI: leaf area index; DMA: dry matter accumulation; FN: fruit number per plant; FW: single fruit weight; WP_C_: crop water productivity; WP_I_: irrigation water use productivity; NPFP: nitrogen partial factor productivity. The shade of color in the legend indicates the strength of correlation. Red circles denote positive correlation, and blue circles denote negative correlation. The size of the circles represents the magnitude of the correlation. *, **, and *** represent significance levels of 0.05, 0.01, and 0.001 respectively.

**Figure 12** presents the correlations among eggplant growth dynamics, dry matter, yield, water productivity, and nitrogen fertilizer use efficiency in 2021 and 2022. PH was strongly and positively correlated (*P* < 0.001) with SD, LAI, and DMA, reflecting a well-coordinated growth pattern in the plants. Furthermore, LAI exhibited a highly significant positive correlation with DMA (*P* < 0.001), indicating that leaf area development significantly contributes to DMA. Similarly, yield exhibited strong positive correlations with both FN and FW, indicating that both FN and FW significantly contribute to yield. *WP_C_* showed positive correlations with yield and FW (*P* < 0.05), and WP_C_ also exhibited a highly significant positive correlation with *WP_I_* (R = 0.99 in 2021 and R = 0.94 in 2022; *P* < 0.001), indicating that WP_C_ and WP_I_ are closely related in water use mechanisms.

### 3.6 Evaluation of integrated strategies for irrigation and nitrogen fertilization

When evaluating core indicators of yield, the W1N2 treatment maintained the highest total yield. In terms of resource utilization efficiency, moderate water deficit management (W1N2, W1N1, and W1N3) maintained high *WP_C_*, while the low nitrogen application rate (W1N1) showed greater advantages in nitrogen fertilizer resource utilization efficiency. However, when determining optimal irrigation volumes and nitrogen fertilizer rates, multiple indicators should be considered alongside yield, including water productivity, nitrogen fertilizer use efficiency, growth status, and DMA. Moreover, different evaluation methods in agricultural production may cause conflicts between high yield and high efficiency. To address this potential, the present study comprehensively employed the TOPSIS method and GRA to evaluate key agronomic traits of eggplant, yield, water productivity, and nitrogen fertilizer use efficiency, with the goal of supporting optimized resource allocation and management strategies.

The comprehensive scores calculated using TOPSIS and GRA are shown in **Table 5**. The TOPSIS results indicated that the W1N2 treatment (2021: 0.9140; 2022: 0.8760) ranked first, while the W2N3 treatment (2021: 0.2380; 2022: 0.2130) ranked last. The GRA results indicated that the W1N2 treatment (2021: 0.9358; 2022: 0.9134) ranked first, while the W2N1 treatment (2021: 0.5444; 2022: 0.5434) ranked last. Although the rankings derived from the two analytical methods exhibited certain discrepancies, the optimal treatments was identical. Considering the data from both years, the top two comprehensive evaluation scores were achieved under moderate nitrogen application (N2), while the lower ranked scores corresponded to moderate water deficit (W2). The W1N2 treatment achieved the highest overall evaluation score. Therefore, the W1N2 treatment yields the optimal comprehensive benefits for eggplant, while the W2N1 and W2N3 treatment represents the least favorable water nitrogen combination.

**Table 5:** Comparison of comprehensive evaluation strategies for eggplant irrigation and nitrogen fertilizer application management under different treatments using TOPSIS and GRA methods.

Year	Treatments	TOPSIS				GRA	
		D+	D-	CI	Rank	Grey Relational Degree	Rank
2021	W0N1	0.197	0.211	0.518	6	0.6389	5
	W0N2	0.084	0.285	0.773	2	0.8358	2
	W0N3	0.163	0.23	0.585	4	0.7840	4
	W1N1	0.189	0.209	0.525	5	0.6361	6
	W1N2	0.03	0.318	0.914	1	0.9358	1
	W1N3	0.141	0.236	0.625	3	0.8051	3
	W2N1	0.255	0.148	0.366	7	0.5444	9
	W2N2	0.222	0.125	0.361	8	0.5954	7
	W2N3	0.303	0.095	0.238	9	0.5616	8
2022	W0N1	0.196	0.216	0.524	6	0.6511	6
	W0N2	0.091	0.284	0.757	2	0.8365	2
	W0N3	0.173	0.228	0.569	4	0.7747	4
	W1N1	0.174	0.216	0.554	5	0.6589	5
	W1N2	0.045	0.316	0.876	1	0.9134	1
	W1N3	0.151	0.231	0.605	3	0.7906	3
	W2N1	0.262	0.141	0.35	8	0.5434	9
	W2N2	0.218	0.131	0.375	7	0.6075	7
	W2N3	0.308	0.083	0.213	9	0.5571	8

TOPSIS represents the technique for order preference by similarity to ideal solution, and GRA represents grey relational analysis. D+ and D- denote positive Euclidean distance and negative Euclidean distance, respectively; CI represents the comprehensive evaluation index.

## 4 Discussion

### 4.1 Effects of different water and nitrogen applications on plant growth

PH, SD, and leaf area are the most direct indicators for assessing eggplant growth status (Guo et al., 2024; Ma et al., 2025). In addition to genetic characteristics, plant growth and development are also closely associated with other factors, such as light, temperature, water, and nutrients (Sun et al., 2025; Zhang et al., 2025). The present findings demonstrated that under the N1 and N3 levels, the W1 treatment reduced PH, SD, and LAI in eggplants during the flowering and fruit set period, while the W2 treatment during the same phenological period significantly (*P* < 0.05) reduced these growth parameters at all nitrogen application levels, with decreases ranging from 14.97–45.62%, 8.13–31.22%, and 22.35–74.41%, respectively. After re-watering during the fruit development stage, the eggplant growth parameters increased, indicating a certain degree of compensatory effect. Moreover, the compensatory growth under the W1 treatment during the flowering and fruit set period was more obvious than that under the W2 treatment. This difference may be attributed to water stress reducing the photosynthetic products of the plant cell walls and protoplasm, which inhibits the elongation and expansion of cells, ultimately leading to growth inhibition, including decreases in PH, SD, and leaf area (Wu et al., 2013). After rehydration, certain metabolites accumulated during water stress may be partly explained by their reutilization for cell wall synthesis and other growth processes once water supply is restored, thereby generating a compensatory growth effect (Huang et al., 2022).

During the flowering and fruit set period in the present study, eggplant PH, SD, and LAI increased with increasing nitrogen rates at the W0 and W1 levels. At the W2 level, these growth parameters initially increased but then decreased with increasing nitrogen rates, peaking at the N2 nitrogen level. These results align with those of previous studies (Wang Y et al., 2024). An increase in irrigation water volume from 75% to 100% of the local typical irrigation quota, coupled with an increase in nitrogen application from 150 kg·ha^-1^ to 250 kg·ha^-1^, resulted in a gradual increase in PH and LAI. Beyond 250 kg·ha^-1^, however, further nitrogen application was detrimental to both parameters, with PH and LAI exhibiting a decreasing trend. This differential impact may be attributed to the dual role of nitrogen fertilization under water-limited conditions. Moderate nitrogen application enhances drought tolerance and mitigates the adverse effects of water deficit on plant growth (Jalil Sheshbahreh et al., 2019; Rostamza et al., 2011). Conversely, excessive nitrogen may impair membrane permeability and suppress the uptake of water and nutrients, consequently inhibiting plant development (Biessy et al., 2025).

### 4.2 Effects of different water and nitrogen applications on plant dry matter

Plant dry matter, derived from photosynthetic activity, serves as an indicator of organic matter accumulation and nutritional status within plants. The accumulation and distribution of plant dry matter are modulated by multiple environmental and physiological factors, including light intensity, air temperature, soil moisture, nutrient availability, and interplant nutrient competition (Zhang et al., 2024; Zheng et al., 2025). In the present study, the DMA of eggplant exhibited a continuous upward trend throughout the growth period, with the cumulative dry matter rate over the entire growth cycle following an S-shaped curve. As water deficit intensified, the accumulation of dry matter in eggplants exhibited a decreasing trend. Compared with adequate irrigation (W0), mild water deficit (W1) caused no significant reduction in DMA during the flowering and fruit set stage, whereas moderate water deficit (W2) led to a significant decrease of 15.80–36.59% during the same stage. Under sufficient (W0) and mild water deficit (W1) conditions, the DMA in eggplants increased with increasing nitrogen rates. At the moderate water deficit (W2) level, DMA initially increased and then decreased, and the peak DMA value was observed at the N2 level. Except for severe water deficiency treatments, the accumulation of assimilates in winter wheat increases with higher water and nitrogen fertilizer rates both before and after flowering. Plants experiencing mild to moderate water stress exhibit improved translocation of preflowering assimilates, whereas severe water deficiency impedes the reallocation of preflowering assimilates (Zhuang et al., 2024). The distribution patterns of dry matter among eggplant organs are modulated by water and nitrogen application treatments. Optimal management of these factors significantly promotes both the accumulation and allocation of photosynthate toward the fruit. The DMA in fruits exhibited an initial increase followed by a decrease as nitrogen application rates increased, peaking at the N2 level before decreasing at higher application levels. Mild water deficit (W1) did not significantly reduce DMA, whereas moderate water deficit (W2) significantly inhibited fruit DMA. Appropriate water deficit and moderate nitrogen fertilization favorably alter the allocation ratio of dry matter to fruit. This occurs because the “source-sink” balance during eggplant growth is modified by water deficit and nitrogen fertilizer (Fang et al., 2022; Fei et al., 2024).

### 4.3 Effects of different water and nitrogen applications on eggplant yield

Under a consistent irrigation level, the medium nitrogen application rate (N2) resulted in the highest FN, which did not differ significantly from that under a high nitrogen application rate (N3). Moreover, moderate nitrogen fertilizer application (N2) significantly increased FW, whereas excessive nitrogen fertilizer application (N3) markedly reduced FW under the same irrigation conditions. The primary factors that affect eggplant yield are FN and FW. At consistent irrigation conditions, an appropriate increase in nitrogen fertilizer (N2) is beneficial for increasing yield, while an excessive increase in nitrogen fertilizer (N3) is not conducive to increasing yield and even poses a risk of a decrease. This may be attributed to moderate nitrogen fertilization enhancing the water uptake capacity of plant roots, leading to increased water potential in leaves, which in turn increases stomatal conductance and promotes photosynthetic metabolism, thereby improving crop yield (Yang et al., 2024). In contrast, excessive nitrogen fertilization increases the LAI of crops, enhances plant transpiration, and reduces photosynthetic rates, thereby inhibiting the formation of crop DMA and yield (Cheng et al., 2022). Under moderate water deficit (W2) conditions in the present study, the yield under the medium nitrogen application (N2) was comparable to that of the low nitrogen application (N1). However, the high nitrogen treatment (N3) exhibited a significant yield reduction of 9.17% (2021) and 7.49% (2022) relative to that of the low nitrogen treatment (N1). This may be due to reduced plant water content under moderate water deficit conditions, where excessive nitrogen application causes osmotic stress to the root system (Gonzalez-Dugo et al., 2010), thereby requiring more energy for osmotic adjustment and leading to decreased transpiration and nutrient uptake capacity (Lahoz et al., 2016), ultimately resulting in lower yields. The W1N2 treatment achieved the highest eggplant yield. Compared with that of the W1N2 treatment, the yield was significantly decreased by 10.03% in 2021 and by 9.73% in 2022 in the W0N3 treatment. These findings indicated that appropriate irrigation and nitrogen application lead to greater improvements in eggplant fruit yield compared with excessive application (Hao et al., 2025; Li et al., 2026). Proper water and nitrogen management ensures that crops obtain optimal resources and enhances plant photosynthesis and carboxylation efficiency, consequently increasing yields (dos Santos Silva et al., 2021; Zhao et al., 2025).

### 4.4 Effects of different water and nitrogen applications on water and nitrogen fertilizer use efficiencies

Optimizing irrigation and nitrogen management in agricultural production is pivotal for enhancing water productivity (Sun et al., 2023). Under a consistent nitrogen application level, mild water deficit during the flowering and fruit set period significantly increased *WP_C_* and *WP_I_*, while moderate water deficit significantly reduced *WP_C_* and *WP_I_*. In contrast to the detrimental effects of both severe water deficit and excessive irrigation, these findings suggested that mild water stress improves *WP_C_*, a finding consistent with a previous study (Liu et al., 2019). Regarding the mean *WP_C_* and *WP_I_* values under the same nitrogen application rate, moderate water deficit did not yield the highest *WP_C_* and *WP_I_* values, indicating that the maximum *WP_C_* and *WP_I_* are not dependent on minimum irrigation rates because severe water stress inhibits plant growth, thereby reducing fruit yield. These results align with prior research conducted on eggplant, potato, and tomato (Badr et al., 2022; Bello et al., 2024a; Li et al., 2023). Under a consistent irrigation level, an appropriate amount of increased nitrogen fertilizer application effectively improved the WP_C_ and WP_I_, aligning with prior research (Dai et al., 2023; Zhao et al., 2023; Zhuang et al., 2024). Adequate nitrogen supply increases crop leaf area, enhances chlorophyll content in leaves, improves photosynthetic processes, and enhances crop drought tolerance through osmotic regulation and plant antioxidant capacity (Jalil Sheshbahreh et al., 2019; Zhang et al., 2017). Adequate nitrogen supply also promotes root growth and water absorption capacity (Du et al., 2017), thereby promoting DMA (Si et al., 2020), increasing yield (Zhang et al., 2016), and consequently improving the *WP_C_* and *WP_I_*. The *WP_C_* and *WP_I_* under the W1N2 treatment were significantly elevated compared with those under other water–nitrogen treatments. This improvement can be explained by the synergistic effect of mild water deficit combined with moderate nitrogen application, which reduces irrigation volume and lowers total plant water consumption while enabling higher eggplant yields, thereby improving the *WP_C_* and *WP_I_* (Yang et al., 2020).

Under consistent irrigation conditions, the NPFP exhibited a decreasing trend as the nitrogen application rates increased. Excessive nitrogen fertilization leads to inefficient consumption, such as nitrogen leaching and volatilization (Hao et al., 2024; Liu et al., 2020). Excessive nitrogen fertilization also increases the ion concentration and osmotic pressure within the root zone, thereby inhibiting root uptake of water and nutrients (Jalil Sheshbahreh et al., 2019). These effects ultimately reduce crop yield and consequently lower the NPFP, which aligns with prior research (Zhuang et al., 2024). The availability of nitrogen in the topsoil is closely related to soil moisture content (Li et al., 2022). Available soil moisture is a prerequisite for determining nitrogen application rates, as soil moisture plays a crucial role in regulating nitrogen cycling within the soil and plant nitrogen uptake (Guo et al., 2012). In the present study, the mean NPFP values at the W0 level was significantly higher than that at the W2 level because an adequate water supply facilitates the efficient transport of nitrogen to plants, thereby enhancing the NPFP (Kunrath et al., 2018). Conversely, when soil moisture is insufficient, nitrogen is difficult for plants to absorb (Kunrath et al., 2020). Although the NPFP perform well under low nitrogen conditions, the yields, WP_C_, and WP_I_ are not optimized under these conditions. Therefore, the most effective irrigation nitrogen management strategy must comprehensively consider both fruit yield and water–nitrogen use efficiency.

### 4.5 Comparative analysis with other dryland crops and study limitations

The water–nitrogen interaction effects observed in this study are broadly consistent with findings reported for other solanaceous vegetable crops. For instance, in tomato, mild water deficit combined with moderate nitrogen fertilization has been shown to improve fruit quality and water use efficiency without significant yield penalty (Bello et al., 2024b; Wang et al., 2015), which parallels our observations in eggplant. Similarly, in pepper, optimal water–nitrogen coupling has been shown to improve photosynthetic efficiency and dry matter partitioning toward reproductive organs (Zhang et al., 2023). These cross-crop consistencies suggest that the “moderate deficit–moderate nitrogen” synergy may represent a general principle for optimizing water and nitrogen management in solanaceous crops under arid and semi-arid conditions. However, differences in root architecture, harvest index, and sensitivity to water stress among these species warrant caution when extrapolating optimal irrigation and nitrogen rates directly across crops.

Despite the valuable insights provided by this study, several limitations should be acknowledged. First, the experiments were conducted using a single eggplant cultivar (Lan Za No. 2), and the responses observed may not be generalizable to other cultivars with different drought tolerance or nitrogen use efficiency characteristics. Second, the study was restricted to two growing seasons (2021 and 2022), which, while providing some inter-annual replication, do not fully account for the variability in climatic conditions that may occur over a longer time series. Third, the present study did not include detailed physiological measurements, such as stomatal conductance, chlorophyll fluorescence, or osmotic adjustment indicators, which would have helped elucidate the mechanistic basis of the observed compensatory growth and water–nitrogen interactions. Future studies incorporating multi-cultivar trials, long-term field observations, and comprehensive physiological and molecular analyses are warranted to further refine irrigation and nitrogen management strategies for eggplant production.

## 5 Conclusions

Under sufficient water supply and mild water deficit conditions, supplemental nitrogen fertilizer promotes eggplant growth. At moderate water deficit levels, an appropriate increase in nitrogen input partially alleviates the adverse impacts of water deficit. However, excessive nitrogen fertilizer application diminishes returns. Under a consistent irrigation level, a moderate increase in nitrogen fertilizer enhances yield by promoting both FN and FW. Conversely, excessive application of nitrogen fertilizer is detrimental to yield increases and may even cause yield reduction. Excessive irrigation and nitrogen fertilizer application both lead to reduced WP_C_ and nitrogen fertilizer use efficiency. In the present study, a comprehensive evaluation was performed employing the TOPSIS method and GRA to assess 10 indicators, covering key agronomic traits, yield, and water–nitrogen use efficiency. The present findings demonstrated that the W1N2 treatment with mild water deficit (soil moisture content at 60–70% field capacity) and medium nitrogen application (nitrogen application rate of 270 kg·ha^-1^) represents the optimal combination of water and nitrogen. Compared with the CK, this treatment increased yield and WP by 32.62% and 37.84% in 2021, and by 35.06% and 42.48% in 2022, respectively. The present study achieved simultaneous optimization of yield and water–nitrogen use efficiency in the Hexi Corridor region, thereby promoting sustainable eggplant production in the Hexi Corridor and offering a reference for similar arid ecosystems.

## Data Availability

The original contributions presented in the study are included in the article/supplementary material. Further inquiries can be directed to the corresponding author.

## References

[B1] Azad N. Behmanesh J. Rezaverdinejad V. Abbasi F. Navabian M. (2020). An analysis of optimal fertigation implications in different soils on reducing environmental impacts of agricultural nitrate leaching. Sci. Rep. 10, 7797. doi: 10.1038/s41598-020-64856-x 32385411 PMC7211014

[B2] Badr M. A. El-Tohamy W. A. Salman S. R. Gruda N. (2022). Yield and water use relationships of potato under different timing and severity of water stress. Agric. Water Manage. 271, 107793. doi: 10.1016/j.agwat.2022.107793 38826717

[B3] Bello A. S. Huda S. Alsafran M. Abu-Dieyeh M. H. Chen Z.-H. Ahmed T. (2024a). Enhancing eggplant (solanum melongena L.) yield and water use efficiency through optimized irrigation and nitrogen practices in open field conditions. J. Agric. Food Res. 18, 101527. doi: 10.1016/j.jafr.2024.101527 38826717

[B4] Bello A. S. Huda A. K. Alsafran M. Jayasena V. Jawaid M. Z. Chen Z. H. . (2024b). Tomato (Solanum lycopersicum) yield response to drip irrigation and nitrogen application rates in open-field cultivation in arid environments. Sci. Hortic. 334, 113298. doi: 10.1016/j.scienta.2024.113298 38826717

[B5] Biessy A. Ciotola M. Cadieux M. Deshaies A. Zboralski A. Khun K. . (2025). Interplay between nitrogen fertilization and plant growth-promoting rhizobacteria impacts lettuce growth under field conditions. Plant Soil. 1–17. doi: 10.1007/s11104-025-07742-7 41523316

[B6] Chen R. Chen X. Li H. Wang J. Guo X. (2025). Evaluating soil water and nitrogen transport, nitrate leaching and soil nitrogen concentration uniformity under sprinkler irrigation and fertigation using numerical simulation. J. Hydrol. 647, 132345. doi: 10.1016/j.jhydrol.2024.132345 38826717

[B7] Cheng M. He J. Wang H. Fan J. Xiang Y. Liu X. . (2022). Establishing critical nitrogen dilution curves based on leaf area index and aboveground biomass for greenhouse cherry tomato: a bayesian analysis. Eur. J. Agron. 141, 126615. doi: 10.1016/j.eja.2022.126615 38826717

[B8] Cheng M. Wang H. Fan J. Xiang Y. Tang Z. Pei S. . (2021). Effects of nitrogen supply on tomato yield, water use efficiency and fruit quality: a global meta-analysis. Sci. Hortic. 290, 110553. doi: 10.1016/j.scienta.2021.110553 38826717

[B9] Coyago-Cruz E. Meléndez-Martínez A. J. Moriana A. Girón I. F. Martín-Palomo M. J. Galindo A. . (2019). Yield response to regulated deficit irrigation of greenhouse cherry tomatoes. Agric. Water Manage. 213, 212–221. doi: 10.1016/j.agwat.2018.10.020 38826717

[B10] Dai Y. Liao Z. Lai Z. Bai Z. Zhang F. Li Z. . (2023). Interactive effects of planting pattern, supplementary irrigation and planting density on grain yield, water-nitrogen use efficiency and economic benefit of winter wheat in a semi-humid but drought-prone region of northwest China. Agric. Water Manage. 287, 108438. doi: 10.1016/j.agwat.2023.108438 38826717

[B11] Ding Y. Shang C. Zhao L. Jin S. Li C. Yin S. . (2025). Effects of irrigation and fertilization management on kiwifruit yield, water use efficiency and quality in China: a meta-analysis. Front. Plant Sci. 16. doi: 10.3389/fpls.2025.1534702 41169724 PMC12568633

[B12] dos Santos Silva A. M. Santos M. V. da Silva L. D. dos Santos J. B. Ferreira E. A. Santos L. D. T. (2021). Effects of irrigation and nitrogen fertilization rates on yield, agronomic efficiency and morphophysiology in tithonia diversifolia. Agric. Water Manage. 248, 106782. doi: 10.1016/j.agwat.2021.106782 38826717

[B13] Du Y. Cao H. Liu S. Gu X. Cao Y. (2017). Response of yield, quality, water and nitrogen use efficiency of tomato to different levels of water and nitrogen under drip irrigation in northwestern China. J. Integr. Agric. 16, 1153–1161. doi: 10.1016/S2095-3119(16)61371-0

[B14] Du Y. Niu W. Gu X. Zhang Q. Cui B. (2018). Water- and nitrogen-saving potentials in tomato production: a meta-analysis. Agric. Water Manage. 210, 296–303. doi: 10.1016/j.agwat.2018.08.035 38826717

[B15] Fan B. Zhang Y. Fenton O. Daly K. Li J. Wang H. . (2024). Irrigation and nitrogen fertiliser optimisation in protected vegetable fields of northern China: achieving environmental and agronomic sustainability. J. Integr. Agric. 23, 1022–1033. doi: 10.1016/j.jia.2023.12.019 38826717

[B16] Fang H. Liu F. Gu X. Chen P. Li Y. P. Li Y. N. (2022). The effect of source–sink on yield and water use of winter wheat under ridge-furrow with film mulching and nitrogen fertilization. Agric. Water Manage. 267, 107616. doi: 10.1016/j.agwat.2022.107616 38826717

[B17] Fei L. Pan Y. Ma H. Guo R. Wang M. Ling N. . (2024). Optimal organic-inorganic fertilization increases rice yield through source-sink balance during grain filling. Field Crops Res. 308, 109285. doi: 10.1016/j.fcr.2024.109285 38826717

[B18] Fernández J. E. Alcon F. Diaz-Espejo A. Hernandez-Santana V. Cuevas M. V. (2020). Water use indicators and economic analysis for on-farm irrigation decision: a case study of a super high density olive tree orchard. Agric. Water Manage. 237, 106074. doi: 10.1016/j.agwat.2020.106074 38826717

[B19] Gonzalez-Dugo V. Durand J.-L. Gastal F. (2010). Water deficit and nitrogen nutrition of crops. A review. Agron. Sustain. Dev. 30, 529–544. doi: 10.1051/agro/2009059 42478355

[B20] Guo S. Wu L. Cao X. Sun X. Cao Y. Li Y. . (2024). Simulation model construction of plant height and leaf area index based on the overground weight of greenhouse tomato: device development and application. Horticulturae 10, 270. doi: 10.3390/horticulturae10030270 30654563

[B21] Guo S. Zhu H. Dang T. Wu J. Liu W. Hao M. . (2012). Winter wheat grain yield associated with precipitation distribution under long-term nitrogen fertilization in the semiarid loess plateau in China. Geoderma 189–190, 442–450. doi: 10.1016/j.geoderma.2012.06.012 38826717

[B22] Hao K. Zhang W. Zhu S. Peng Y. Zhong Y. Jie F. . (2025). Alternate partial root-zone irrigation combined with nitrogen fertilizer: An adaptive surge root irrigation and nitrogen strategy to improve apple yield, water-nitrogen use efficiency and fruit quality. Agric. Water Manage. 308, 109296. doi: 10.1016/j.agwat.2025.109296 38826717

[B23] Hao Y. Zheng T. Zheng X. Liu L. Jiang S. Cao M. (2024). The impact of dissolved organic nitrogen (DON) retention in the vadose zone on nitrogen leaching losses. Chemosphere 366, 143449. doi: 10.1016/j.chemosphere.2024.143449 39362379

[B24] He Z. Li M. Cai Z. Zhao R. Hong T. Yang Z. (2021). Optimal irrigation and fertilizer amounts based on multi-level fuzzy comprehensive evaluation of yield, growth and fruit quality on cherry tomato. Agric. Water Manage. 243, 106360. doi: 10.1016/j.agwat.2020.106360 38826717

[B25] Hou D. Liu K. Liu S. Li J. Tan J. Bi Q. (2024). Enhancing root physiology for increased yield in water-saving and drought-resistance rice with optimal irrigation and nitrogen. Front. Plant Sci. 15. doi: 10.3389/fpls.2024.1370297 38779071 PMC11109435

[B26] Hou Y. Wang Z. Ding H. Li W. Wen Y. Zhang J. (2019). Evaluation of suitable amount of water and fertilizer for mature grapes in drip irrigation in extreme arid regions. Sustainability 11, 2063. doi: 10.3390/su11072063 30654563

[B27] Hu J. Gettel G. Fan Z. Lv H. Zhao Y. Yu Y. . (2021). Drip fertigation promotes water and nitrogen use efficiency and yield stability through improved root growth for tomatoes in plastic greenhouse production. Agric. Ecosyst. Environ. 313, 107379. doi: 10.1016/j.agee.2021.107379 38826717

[B28] Huang H. Cao Y. Xin K. Liang R. Chen Y. Qi J. (2022). Morphological and physiological changes in artemisia selengensis under drought and after rehydration recovery. Front. Plant Sci. 13. doi: 10.3389/fpls.2022.851942 35991406 PMC9389366

[B29] Jalil Sheshbahreh M. Movahhedi Dehnavi M. Salehi A. Bahreininejad B. (2019). Effect of irrigation regimes and nitrogen sources on biomass production, water and nitrogen use efficiency and nutrients uptake in coneflower (eChinacea purpurea L.). Agric. Water Manage. 213, 358–367. doi: 10.1016/j.agwat.2018.10.011 38826717

[B30] Karimi A. Kazemi M. Samani S. A. Simal-Gandara J. (2021). Bioactive compounds from by-products of eggplant: functional properties, potential applications and advances in valorization methods. Trends Food Sci. Technol. 112, 518–531. doi: 10.1016/j.tifs.2021.04.027 38826717

[B31] Kunrath T. R. Lemaire G. Sadras V. O. Gastal F. (2018). Water use efficiency in perennial forage species: interactions between nitrogen nutrition and water deficit. Field Crops Res. 222, 1–11. doi: 10.1016/j.fcr.2018.02.031 38826717

[B32] Kunrath T. R. Lemaire G. Teixeira E. Brown H. E. Ciampitti I. A. Sadras V. O. (2020). Allometric relationships between nitrogen uptake and transpiration to untangle interactions between nitrogen supply and drought in maize and sorghum. Eur. J. Agron. 120, 126145. doi: 10.1016/j.eja.2020.126145 38826717

[B33] Lahoz I. Pérez-de-Castro A. Valcárcel M. Macua J. I. Beltrán J. Roselló S. . (2016). Effect of water deficit on the agronomical performance and quality of processing tomato. Sci. Hortic. 200, 55–65. doi: 10.1016/j.scienta.2015.12.051 38826717

[B34] Li H. Hou X. Bertin N. Ding R. Du T. (2023). Quantitative responses of tomato yield, fruit quality and water use efficiency to soil salinity under different water regimes in northwest China. Agric. Water Manage. 277, 108134. doi: 10.1016/j.agwat.2022.108134 38826717

[B35] Li Y. Huang G. Chen Z. Xiong Y. Huang Q. Xu X. . (2022). Effects of irrigation and fertilization on grain yield, water and nitrogen dynamics and their use efficiency of spring wheat farmland in an arid agricultural watershed of northwest China. Agric. Water Manage. 260, 107277. doi: 10.1016/j.agwat.2021.107277 38826717

[B36] Li X. Li S. Qiang X. Yu Z. Sun Z. Wang R. . (2024). Effects of water and nitrogen regulation on apple tree growth, yield, quality, and their water and nitrogen utilization efficiency. Plants. 13, 2404. doi: 10.3390/plants13172404 39273888 PMC11396910

[B37] Li S. Li H. J. Yang J. Si Z. Zhou T. Ren R. . (2026). Integrating water and nitrogen optimization to enhance wheat yield and macronutrients absorption under high-low seedbed cultivation. Irrig. Sci. 44, 28. doi: 10.1007/s00271-025-01056-3 30311153

[B38] Li H. Liu H. Gong X. Li S. Pang J. Chen Z. . (2021). Optimizing irrigation and nitrogen management strategy to trade off yield, crop water productivity, nitrogen use efficiency and fruit quality of greenhouse grown tomato. Agric. Water Manage. 245, 106570. doi: 10.1016/j.agwat.2020.106570 38826717

[B39] Liu H. Li H. Ning H. Zhang X. Li S. Pang J. . (2019). Optimizing irrigation frequency and amount to balance yield, fruit quality and water use efficiency of greenhouse tomato. Agric. Water Manage. 226, 105787. doi: 10.1016/j.agwat.2019.105787 38826717

[B40] Liu X. Zhang Y. Jiang Z. Yue X. Liang J. Yang Q. . (2023). Micro-moistening irrigation combined with bio-organic fertilizer: an adaptive irrigation and fertilization strategy to improve soil environment, edible rose yield, and nutritional quality. Ind. Crops Prod. 196, 116487. doi: 10.1016/j.indcrop.2023.116487 38826717

[B41] Liu L. Zhang X. Xu W. Liu X. Li Y. Wei J. . (2020). Ammonia volatilization as the major nitrogen loss pathway in dryland agro-ecosystems. Environ. pollut. 265, 114862. doi: 10.1016/j.envpol.2020.114862 32497822

[B42] Lu J. Hu T. Zhang B. Wang L. Yang S. Fan J. . (2021). Nitrogen fertilizer management effects on soil nitrate leaching, grain yield and economic benefit of summer maize in northwest China. Agric. Water Manage. 247, 106739. doi: 10.1016/j.agwat.2021.106739 38826717

[B43] Lv H. Lin S. Wang Y. Lian X. Zhao Y. Li Y. . (2019). Drip fertigation significantly reduces nitrogen leaching in solar greenhouse vegetable production system. Environ. pollut. 245, 694–701. doi: 10.1016/j.envpol.2018.11.042 30500748

[B44] Ma M. Zhao J. Yang T. Liu F. Yuan Y. Ma S. . (2025). Estimating comprehensive growth index for drip-irrigated spring maize in junggar basin via satellite imagery and machine learning. Agric. Water Manage. 318, 109651. doi: 10.1016/j.agwat.2025.109651 38826717

[B45] Muhirwa F. Li L. Laspidou C. (2025). Global ecosystem sustainability indexing and patterns in the success of SDGs of water, energy and food security. J. Cleaner Prod. 516, 145830. doi: 10.1016/j.jclepro.2025.145830 38826717

[B46] Mwinuka P. R. Mbilinyi B. P. Mbungu W. B. Mourice S. K. Mahoo H. F. Schmitter P. (2021). Optimizing water and nitrogen application for neglected horticultural species in tropical sub-humid climate areas: a case of african eggplant (solanum aethiopicum L.). Sci. Hortic. 276, 109756. doi: 10.1016/j.scienta.2020.109756 38826717

[B47] Rostamza M. Chaichi M.-R. Jahansouz M.-R. Alimadadi A. (2011). Forage quality, water use and nitrogen utilization efficiencies of pearl millet (pennisetum americanum L.) grown under different soil moisture and nitrogen levels. Agric. Water Manage. 98, 1607–1614. doi: 10.1016/j.agwat.2011.05.014 38826717

[B48] Scherger L. E. Zanello V. Lafont D. Lexow C. (2021). Modeling fate and transport of ammonium, nitrite, and nitrate in a soil contaminated with large dose of urea. Environ. Earth Sci. 80, 539. doi: 10.1007/s12665-021-09814-0 30311153

[B49] Si Z. Zain M. Mehmood F. Wang G. Gao Y. Duan A. (2020). Effects of nitrogen application rate and irrigation regime on growth, yield, and water-nitrogen use efficiency of drip-irrigated winter wheat in the north China plain. Agric. Water Manage. 231, 106002. doi: 10.1016/j.agwat.2020.106002 38826717

[B50] Sun W. He Q. Zhou G. Song Y. (2025). Response of grain quality to plant growth dynamics in summer maize as influenced by sowing dates and weather factors. Ind. Crops Prod. 231, 121210. doi: 10.1016/j.indcrop.2025.121210 38826717

[B51] Sun Y. Hu K. Fan Z. Wei Y. Lin S. Wang J. (2013). Simulating the fate of nitrogen and optimizing water and nitrogen management of greenhouse tomato in north China using the EU-Rotate_N model. Agric. Water Manage. 128, 72–84. doi: 10.1016/j.agwat.2013.06.016 38826717

[B52] Sun L. Li B. Yao M. Niu D. Gao M. Mao L. . (2023). Optimising water and nitrogen management for greenhouse tomatoes in northeast China using EWM-TOPSIS-AISM model. Agric. Water Manage. 290, 108579. doi: 10.1016/j.agwat.2023.108579 38826717

[B53] Thompson R. (2023). Editorial note on terms for soil analyses, nutrient content of fertilizers and Nitrogen Use Efficiency. Agric. Water Manage. 289, 108547. doi: 10.1016/j.agwat.2023.108547 38826717

[B54] Toppino L. Barchi L. Mercati F. Acciarri N. Perrone D. Martina M. . (2020). A new intra-specific and high-resolution genetic map of eggplant based on a RIL population, and location of QTLs related to plant anthocyanin pigmentation and seed vigour. Genes. 11, 745. doi: 10.3390/genes11070745 32635424 PMC7397344

[B55] Wang C. Gu F. Chen J. Yang H. Jiang J. Du T. . (2015). Assessing the response of yield and comprehensive fruit quality of tomato grown in greenhouse to deficit irrigation and nitrogen application strategies. Agric. Water Manage. 161, 9–19. doi: 10.1016/j.agwat.2015.07.010 38826717

[B56] Wang H. Li G. Huang W. Li Z. Wang X. Gao Y. (2024). Compensation of cotton yield by nitrogen fertilizer in non-mulched fields with deficit drip irrigation. Agric. Water Manage. 298, 108850. doi: 10.1016/j.agwat.2024.108850 38826717

[B57] Wang Y. Pan X. Deng H. Li M. Zhao J. Yang J. (2024). Effect of water and nitrogen coupling regulation on the growth, physiology, yield, and quality attributes of isatis tinctoria L. in the oasis irrigation area of the hexi corridor. Agronomy. 14, 2187. doi: 10.3390/agronomy14102187 30654563

[B58] Wu Y. Si W. Yan S. Wu L. Zhao W. Zhang J. . (2023). Water consumption, soil nitrate-nitrogen residue and fruit yield of drip-irrigated greenhouse tomato under various irrigation levels and fertilization practices. Agric. Water Manage. 277, 108092. doi: 10.1016/j.agwat.2022.108092 38826717

[B59] Wu Y. Zhao Z. Wang W. Ma Y. Huang X. (2013). Yield and growth of mature pear trees under water deficit during slow fruit growth stages in sparse planting orchard. Sci. Hortic. 164, 189–195. doi: 10.1016/j.scienta.2013.09.025 38826717

[B60] Xu G. Lu D. Wang H. Li Y. (2018). Morphological and physiological traits of rice roots and their relationships to yield and nitrogen utilization as influenced by irrigation regime and nitrogen rate. Agric. Water Manage. 203, 385–394. doi: 10.1016/j.agwat.2018.02.033 38826717

[B61] Xu R. Shi W. Kamran M. Chang S. Jia Q. Hou F. (2023). Grass-legume mixture and nitrogen application improve yield, quality, and water and nitrogen utilization efficiency of grazed pastures in the loess plateau. Front. Plant Sci. 14. doi: 10.3389/fpls.2023.1088849 36814753 PMC9940662

[B62] Yang H. Du T. Qiu R. Chen J. Wang F. Li Y. . (2017). Improved water use efficiency and fruit quality of greenhouse crops under regulated deficit irrigation in northwest China. Agric. Water Manage. 179, 193–204. doi: 10.1016/j.agwat.2016.05.029 38826717

[B63] Yang D. Li S. Kang S. Du T. Guo P. Mao X. . (2020). Effect of drip irrigation on wheat evapotranspiration, soil evaporation and transpiration in northwest China. Agric. Water Manage. 232, 106001. doi: 10.1016/j.agwat.2020.106001 38826717

[B64] Yang T. Zhao J. Hong M. Ma M. (2024). Appropriate water and nitrogen supply regulates the dynamics of nitrogen translocation and thereby enhancing the accumulation of nitrogen in maize grains. Agric. Water Manage. 306, 109160. doi: 10.1016/j.agwat.2024.109160 38826717

[B65] Ye T. Ma J. Zhang P. Shan S. Liu L. Tang L. . (2022). Interaction effects of irrigation and nitrogen on the coordination between crop water productivity and nitrogen use efficiency in wheat production on the north China plain. Agric. Water Manage. 271, 107787. doi: 10.1016/j.agwat.2022.107787 38826717

[B66] Ye T. Zhang Y. Xuan J. Wang X. Li Y. Xu J. . (2024). Development of a novel critical nitrogen concentration–cumulative transpiration curve for optimizing nitrogen management under varying irrigation conditions in winter wheat. Crop J. 12, 1242–1251. doi: 10.1016/j.cj.2024.06.008 38826717

[B67] Zamljen T. Lojen S. Slatnar A. Zupanc V. (2022). Effect of deficit irrigation on nitrogen accumulation and capsaicinoid content in capsicum plants using the isotope 15N. Agric. Water Manage. 260, 107304. doi: 10.1016/j.agwat.2021.107304 38826717

[B68] Zhang F. Chen M. Xing Y. Wang X. (2025). Appropriate nitrogen application under ridge-furrow plastic film mulching planting optimizes spring maize growth characteristics by improving soil quality in the loess plateau of China. Agric. Water Manage. 307, 109259. doi: 10.1016/j.agwat.2024.109259 38826717

[B69] Zhang Y. Wang J. Gong S. Xu D. Sui J. (2017). Nitrogen fertigation effect on photosynthesis, grain yield and water use efficiency of winter wheat. Agric. Water Manage. 179, 277–287. doi: 10.1016/j.agwat.2016.08.007 38826717

[B70] Zhang H. Wang Y. Yu S. Zhou C. Li F. Chen X. . (2023). Plant photosynthesis and dry matter accumulation response of sweet pepper to water–nitrogen coupling in cold and arid environment. Water 15, 2134. doi: 10.3390/w15112134 30654563

[B71] Zhang R. Yang D. Li S. Chen J. Hu D. Guo H. . (2024). Coupling of environmental factors and growth stages in simulation of maize biomass allocation. Plant Soil 499, 329–347. doi: 10.1007/s11104-022-05794-7 30311153

[B72] Zhang D. Yao P. Zhao N. Yu C. Cao W. Gao Y. (2016). Contribution of green manure legumes to nitrogen dynamics in traditional winter wheat cropping system in the loess plateau of China. Eur. J. Agron. 72, 47–55. doi: 10.1016/j.eja.2015.09.012 38826717

[B73] Zhao B. Niu X. Ata-Ul-Karim S. T. Wang L. Duan A. Liu Z. . (2020). Determination of the post-anthesis nitrogen status using ear critical nitrogen dilution curve and its implications for nitrogen management in maize and wheat. Eur. J. Agron. 113, 125967. doi: 10.1016/j.eja.2019.125967 38826717

[B74] Zhao L. Wang G. Tang Q. Song Z. Luo H. Li Y. (2025). Nitrogen supply under mulched drip irrigation increases the rice yield by improving the photosynthetic nitrogen distribution strategy and promoting biomass accumulation. Agric. Water Manage. 320, 109839. doi: 10.1016/j.agwat.2025.109839 38826717

[B75] Zhao K. Wang H. Wu J. Liu A. Huang X. Li G. . (2023). One-off irrigation improves water and nitrogen use efficiency and productivity of wheat as mediated by nitrogen rate and tillage in drought-prone areas. Field Crops Res. 295, 108898. doi: 10.1016/j.fcr.2023.108898 38826717

[B76] Zheng Q. Song P. Feng J. Wang X. Han S. Zhang K. . (2025). Optimizing water-fertilizer synergy in vegetable-type brassica napus L.: effects of split fertilization on crop performance and resource use efficiency. Agric. Water Manage. 322, 110016. doi: 10.1016/j.agwat.2025.110016 38826717

[B77] Zhou X. Du C. Li H. Sun Z. Chen Y. Gao Z. . (2024). Optimization of inter-seasonal nitrogen allocation increases yield and resource-use efficiency in a water-limited wheat–maize cropping system in the north China plain. Crop J. 12, 907–914. doi: 10.1016/j.cj.2024.03.010 38826717

[B78] Zhou J. Luo M. Ning P. Gong S. Cheng X. Huang X. (2025). Synergistic effects of nitrogen application enhance drought resistance in machilus yunnanensis seedlings. Plants. 14, 3194. doi: 10.3390/plants14203194 41157751 PMC12566645

[B79] Zhou C. Zhang H. Li F. Wang Y. Wang Y. Wang Z. (2022). Deficit mulched drip irrigation improved yield and quality while reduced water consumption of isatis indigotica in a cold and arid environment. Front. Plant Sci. 13, 1013131. doi: 10.3390/w15112134 36247605 PMC9563244

[B80] Zhou C. Zhang H. Yu S. Chen X. Li F. Wang Y. . (2023). Optimizing water and nitrogen management strategies to improve their use efficiency, eggplant yield and fruit quality. Front. Plant Sci. 14. doi: 10.3389/fpls.2023.1211122 37767295 PMC10519791

[B81] Zhu J. Shangguan Y. Dai H. Guo Q. Li C. Yu L. (2025). Application of nano-selenium to tea plants under various nitrogen circumstances improves tea quality and drought resistance. Plant Stress 18, 101139. doi: 10.1016/j.stress.2025.101139 38826717

[B82] Zhuang T. Ata-UI-Karim S. T. Zhao B. Liu X. Tian Y. Zhu Y. . (2024). Investigating the impacts of different degrees of deficit irrigation and nitrogen interactions on assimilate translocation, yield, and resource use efficiencies in winter wheat. Agric. Water Manage. 304, 109089. doi: 10.1016/j.agwat.2024.109089 38826717

